# Comprehensive integrative profiling of upper tract urothelial carcinomas

**DOI:** 10.1186/s13059-020-02230-w

**Published:** 2021-01-04

**Authors:** Xiaoping Su, Xiaofan Lu, Sehrish Khan Bazai, Eva Compérat, Roger Mouawad, Hui Yao, Morgan Rouprêt, Jean-Philippe Spano, David Khayat, Irwin Davidson, Nizar N. Tannir, Fangrong Yan, Gabriel G. Malouf

**Affiliations:** 1 Department of Bioinformatics and Computational Biology, The University of Texas MD Anderson Cancer Center, Houston, TX USA; 2 State Key Laboratory of Natural Medicines, Research Center of Biostatistics and Computational Pharmacy, China Pharmaceutical University, Nanjing, 210009 China; 3 Department of Cancer and Functional Genomics, Institute of Genetics and Molecular and Cellular Biology, CNRS/INSERM/UNISTRA, 67400 Illkirch, France; 4 Equipe Labellisée Ligue Nationale Contre le Cancer, Paris, France; 5 Department of Pathology, GRC No. 5, Predictive Onco Uro, AP-HP, Hôpital Tenon, Sorbonne University, Paris, France; 6 Department of Medical Oncology, AP-HP, Hôpital Pitié-Salpêtrière, Sorbonne University, Paris, France; 7 Department of Urology, GRC No. 5, Predictive Onco Uro, AP-HP, Hôpital Pitié-Salpêtrière, Sorbonne University, Paris, France; 8 Department of Genitourinary Medical Oncology, The University of Texas MD Anderson Cancer Center, Houston, TX USA; 9 Department of Medical Oncology, Institut de Cancérologie de Strasbourg-Europe, Strasbourg, France; 10 Centre Hospitalier Régional Universitaire de Strasbourg, Strasbourg, France

**Keywords:** Upper tract urothelial carcinomas, *ZFP36L1*, DNA methylation, SWI/SNF gene mutations, Sequencing, Immunity, Epigenetics

## Abstract

**Background:**

Crosstalk between genetic, epigenetic, and immune alterations in upper tract urothelial carcinomas and their role in shaping muscle invasiveness and patient outcome are poorly understood.

**Results:**

We perform an integrative genome- and methylome-wide profiling of diverse non-muscle-invasive and muscle-invasive upper tract urothelial carcinomas. In addition to mutations of *FGFR3* and *KDM6A*, we identify *ZFP36L1* as a novel, significantly mutated tumor suppressor gene. Overall, mutations of *ZFP36* family genes (*ZFP36*, *ZFP36L1*, and *ZFP36L2*) are identified in 26.7% of cases, which display a high mutational load. Unsupervised DNA methylation subtype classification identifies two epi-clusters associated with distinct muscle-invasive status and patient outcome, namely, EpiC-low and EpiC-high. While the former is hypomethylated, immune-depleted, and enriched for *FGFR3*-mutated, the latter is hypermethylated, immune-infiltrated, and tightly associated with somatic mutations of *SWI/SNF* genes.

**Conclusions:**

Our study delineates for the first time the key role for convergence between genetic and epigenetic alterations in shaping clinicopathological and immune upper tract urothelial carcinoma features.

## Introduction

Urothelial carcinoma is considered the fifth most common cancer in Western countries, and it is divided at the pathological level into two groups: non-muscle-invasive (NMI) and muscle-invasive (MI) tumors, according to the level of invasion of the detrusor muscle. NMI represents 75% of tumors, while MI represents the latter 25% [1]. NMI tumors are costly to treat as they often recur, and 10–15% of those patients progress to an MI state [1]. Upper tract urothelial carcinomas represent 5–10% among all urothelial carcinomas, and they can arise within the ureter or the renal pelvis, which are derived from different embryonic tissues as compared to the bladder urothelium [2, 3]. Such differences might dictate whether patients with UTUC display a higher incidence of invasive disease at diagnosis as compared to patients with bladder carcinomas [3].

While genome-wide genetic alterations of muscle-invasive bladder carcinomas have been extensively studied by The Cancer Genome Atlas (TCGA) project and others, genetic alterations occurring in UTUC are limited [4, 5]. Recent studies using whole-exome sequencing and/or targeted sequencing of UTUC samples identified recurrent mutations of genes known to be altered in bladder carcinomas, although with different frequencies (e.g., *HRAS*) [6, 7]. At the transcriptomic level, UTUCs were mostly found to be luminal papillary and displayed T cell-depleted immune contexture, possibly related to the FGFR3 overexpression [5]. However, to our knowledge, epigenetic alterations in UTUC, in particular, DNA methylation, and their crosstalk with genetic and transcriptomic clinicopathological tumor features are unknown.

Although the epigenetic alterations of MI bladder carcinomas have been extensively studied by TCGA and other groups, comparative analysis of epigenetic alterations in MI relative to NMI bladder carcinomas has been limited, using often selected cancer-related genes or supervised analysis [8]. In fact, the recent remarkable study exploring genome-wide alterations of NMI bladder carcinomas did not include DNA methylation profiling [9].

To fill this knowledge gap, we decided to investigate the putative contribution of both genetic and epigenetic alterations in dictating muscle invasiveness, a key predictor of poor outcome in UTUC patients. We identified different key findings, including the discovery of novel mutations affecting the zinc-finger RNA-binding protein *ZFP36L1* in 20% of cases, a rate by far the highest among cancer subtypes profiled to date. In addition, we identified two methylome clusters, EpiC-low and EpiC-high, associated with distinct clinicopathological and genetic tumor features, as well as patient survival. While the EpiC-low cluster was hypermethylated, immune-inflamed, enriched in MI cases, and harbored a high rate of somatic mutations of *SWI/SNF* genes, the EpiC-high cluster was hypomethylated, immune-desert, enriched for NMI cases, and harbored a high rate of *FGFR3* mutations. These data pave the way for therapeutic interventions in the most threatening subgroup of UTUC, providing a rational for personalizing therapies in this setting.

## Results

### Samples, clinical data, and analytic approach

Forty fresh-frozen surgically resected primary UTUCs, including 20 NMI and 20 MI cases, were collected retrospectively from the pathology biobank at Pitié-Salpêtrière Hospital and re-evaluated histopathologically by one expert pathologist (E.C.). According to the European Association of Urology guidelines, all samples had been tested for microsatellite instability status; all were microsatellite stable. Overall, DNA and RNA of good quality and quantity were obtained for 40 and 20 cases, respectively. Matched germline DNA from adjacent bladder tissues were also collected in 30 cases. Whole-exome sequencing (WES) was performed for 30-paired UTUCs and adjacent normal tissues. Targeted *FGFR3* sequencing was performed for 35 cases. RNA sequencing was performed on 20 UTUC cases; in addition, DNA methylation was analyzed using Infinium EPIC arrays on 35 UTUC cases and 8 normal adjacent bladder tissues. Detailed clinical and pathologic characteristics of the cohort are reported in Additional file 1: Table S1. The median follow-up time for the 40 patients was 53 months (range 2–98); 18 patients had recurred, and 12 patients have died from disease progression at the last follow-up. Among the clinical variables, muscle invasion status, age, gender, pathological stage, localization, and grade were not associated with progression-free survival or with overall survival (Additional file 1: Table S2).

### Landscape of somatic mutations and focal copy number alterations

**Fig. 1:**
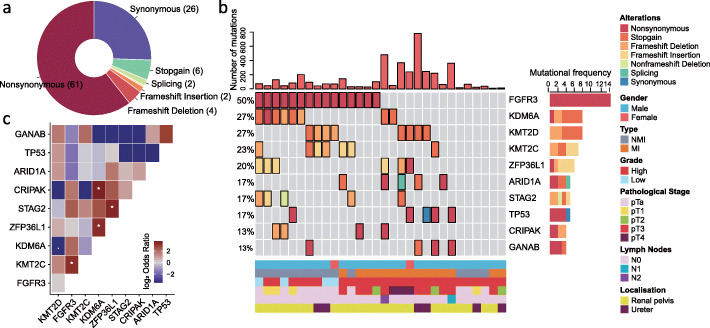
Genetic landscape of somatic mutations identified by whole-exome sequencing of 30 upper tract urothelial carcinomas (UTUC), including 15 muscle-invasive tumors and 15 non-muscle-invasive tumors. **a** Distribution of somatic mutations showing that non-synonymous mutations are the most frequent type. **b** OncoPrint depicting mutational load and most frequently mutated genes identified (≥ 10% of cases) in the whole UTUC cohort. **c** Heatmap for genes with mutual exclusivity or co-occurrence in the whole UTUC cohort. Stars refer to correlations that are statistically significant

The 30 UTUC samples studied by WES included 15 NMI and 15 MI cases. We identified 4239 putative somatic mutations, including 2569 missense mutations, 1081 silent mutations, and 272 indels, with an average of 2.9 ± 3.5 mutations per megabase (Additional file 1: Table S3). Non-synonymous single-nucleotide variants were the most frequent mutations identified (61%) (Fig. 1a). The median mutational load per megabase was 1.5 (range 0.3–15.7). Using MutSigCV, significantly mutated genes (SMGs) (false discovery rate [FDR] < 0.05) were *FGFR3* (50%, FDR = 3.15 × 10^−3^), *KDM6A* (27%, FDR = 1.03 × 10^−3^), and *ZFP36L1* (20%, FDR = 2.62 × 10^−3^) (Fig. 1b, Additional file 1: Table S4). *ZFP36L1* was not previously reported to be mutated in UTUC; as a member of ZFP36 family genes, *ZFP36L1* is a zinc-finger RNA-binding protein that regulates several cytoplasmic AU-rich element (ARE)-containing mRNA transcripts by favoring their poly (A) tail removal or deadenylation, leading to the attenuation of protein synthesis. Mutations of *FGFR3*, *KDM6A*, and *ZFP36L1* alterations were all verified by Sanger sequencing. Other frequent mutations (> 10%) with a trend of significance (*P* < 0.05, FDR > 0.05) involved *MLL2* (*P* = 0.037), *KMT2C* (*P* = 0.02), *STAG2* (*P* = 8.3 × 10^−7^), *ARID1A* (*P* = 0.01), *TP53* (*P* = 0.018), *CRIPAK* (*P* = 0.0001), and *GANAB* (*P* = 0.04) (Fig. 1c). Mutations of *FGFR3* and *KMT2C* co-occur mutually (log_2_ odd ratio = 3.1, *P* = 0.04; Fig. 1c, Additional file 1: Table S5); this was also validated in the MSKCC UTUC cohort (log_2_ odd ratio = 2.9, *P* < 0.001; FDR < 0.001) [7]. In addition, we observed a mutual co-occurrence of mutations affecting *ZFP36L1* with both *KDM6A* (log_2_ odd ratio = 3.1; *P* = 0.03) and *STAG2* (log_2_ odd ratio = 3.29, *P* = 0.04; Additional file 1: Table S5). Mutations of *MLL2* (*P* = 0.04), *ARID1A* (*P* = 0.01), and *GANAB* (*P* = 0.02) showed significant association with a higher mutation load, as well as a tendency for *ZFP36L1* (*P* = 0.06).

Among genes with mutations arising in > 10% of samples, only *FGFR3* mutations were significantly associated with improved overall survival (OS) (*P* = 0.005) and progression-free survival (PFS) at distant sites (*P* = 0.007). We then looked for an association between gene mutations and muscle-invasive status; as expected, we found that *FGFR3* mutations were enriched in NMI as compared to MI tumors (73% vs 27%; *P* = 0.027, FDR = 0.2); conversely, *ARID1A* mutations were enriched in MI tumors (33% vs 0%; *P* = 0.042, FDR = 0.2; Additional file 1: Table S6).

We then analyzed statistically significant focal copy number variation (CNV) changes using the GISTIC algorithm. Considering focal peaks, 1p36.33, 9p21.3, and 11p15.1 were lost (Additional file 2: Fig. S1a). Notably, 9p21.3 encompass *CDKN2A* and *CDKN2B* tumor suppressor genes. The analysis revealed six additional regions with gains at a significant frequency at 1q23.3, 6p21.33, 8p11.23, 8q22.3, 12q15, and 19q12 regions. Several of these events involve known cancer-related oncogenes, such as *YWHAZ* (8q22.3), *MDM2* (12q15), and *CCNE1* (19q12) (Additional file 2: Fig. S1b). We then analyzed the association between CNV, somatic mutations, and clinicopathological tumor features and somatic mutations. We identified significant enrichment in MI cases for 8q22.3 gain, 12q15 gain, and 11p15.11 loss (Additional file 2: Fig. S1c). We also observed mutual exclusivity of 8q22.3 gain with *FGFR3* mutations (*P* = 0.03), as well as mutual occurrence with *TP53* mutations (*P* = 0.02) (Additional file 2: Fig. S1d).

### Mutational frequency comparison between UTUC and BLCA according to muscle invasiveness status

We hypothesized that UTUC and BLCA may show different mutational frequencies according to muscle invasiveness status. To compare mutational frequency regarding NMI, we retrieved mutation data of 24 NMI-BLCA from the Hurst cohort and compared it with our NMI-UTUCs (*n* = 15), and we found *KMT2C* (*P* = 0.08) was more likely to mutate in NMI-UTUCs (n = 15) whereas *PIK3CA* mutations were significantly enriched in NMI-BLCAs (*P* = 0.0001) (Additional file 2: Fig. S2a). We then compared the most frequently mutated genes between MI-UTUC (*n* = 15) and TCGA MI-BLCA (*n* = 412). We found that MI-UTUC showed significantly more mutations of *GANAB* (*P* = 0.021), *CRIPAK* (*P* = 0.022), and *ZFP36L1* (*P* = 0.1) (Additional file 2: Fig. S2b).

### Prevalence of mutations affecting *ZFP36* genes family

**Fig. 2:**
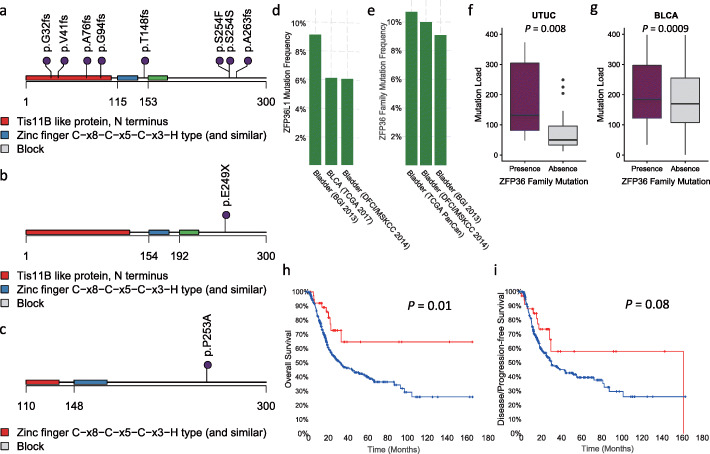
Distribution of mutations affecting *ZFP36* family genes in UTUC and several public bladder carcinoma datasets. Lollipops represent mutations of **a** *ZFP36L1*, **b** *ZFP36L2*, and **c** *ZFP36* genes. **d** Distribution of mutation frequency of *ZFP36L1* in different public bladder carcinoma cohorts using the cBioPortal database. **e** Distribution of mutation frequency of *ZFP36* family in different bladder carcinoma cohorts using the cBioPortal database. **f** Box plot representing the mutational load in UTUC samples according to *ZFP36* family gene mutations. **g** Box plot representing mutational load in bladder carcinoma TCGA cohort according to *ZFP36* family mutations. **h** Kaplan-Meier curves for overall survival of patients with bladder carcinomas in TCGA cohort according to *ZFP36* family gene mutations status. **i** Kaplan-Meier curves for progression-free survival of patients with bladder carcinomas in TCGA cohort according to *ZFP36* family mutations

As *ZFP36L1* mutations have not been previously reported to be altered in UTUC, we thus investigated their significance. Overall, eight mutations were identified in a total of six cases. Strikingly, six out of eight *ZFP36L1* mutations were frameshift insertions or deletions, and two were a non-synonymous mutation predicted to be deleterious by Poly2phen and SIFT (Fig. 2a). When we explored mutations of other members of the *ZFP36* family, we identified one additional UTUC case with a *ZFP36L2* stop-gain mutation (E249X) and another with a deleterious *ZFP36* (p.P253A) mutation, both already existing in COSMIC (Fig. 2b, c). Notably, *ZFP36L2* E249X was identified as a hotspot for mutations in the bladder TCGA, consolidating our findings about the relevance of this mutation in urothelium carcinogenesis (Additional file 2: Fig. S3a). Thus, overall mutations of *ZFP36* family genes represent 26.7% (*n* = 8/30) among all UTUC cases. Mutations of *ZFP36* family genes were not associated with clinicopathological tumor features and patient overall survival (not shown).

### Frequency of *ZFP36* family gene mutations across cancer subtypes

We then decided to analyze the frequency of *ZFP36L1* mutations in 10,967 cancer samples related to 32 different histopathological cancer subtypes analyzed by TGCA. Overall, 120 (1.09%) samples were identified, and the highest frequency was observed in bladder cancer (*n* = 30/410; 7.3%) indicating the importance of this gene both in UTUC and in bladder carcinogenesis (Additional file 2: Fig. S3b). When we combined mutations of *ZFP36L2* and *ZFP36* to *ZFP36L1*, the frequency of *ZFP36* gene family mutations reached 3% (*n* = 297) in all TCGA cohorts, with the highest frequencies observed in bladder cancers (Additional file 2: Fig. S3c). Strikingly, *ZFP36L1* mutations were consistently altered in three independent studies exploring the genetic landscape of bladder carcinomas (TCGA, DFCI, and BGI), with frequencies ranging from 6 to 8.5% (Fig. 2d). The frequency of *ZFP36* gene family mutations ranged between 9.1 and 10%, among the three bladder carcinoma cohorts (Fig. 2e); notably, mutations of *ZFP36L1* and *ZFP36L2* were mutually co-occurring (*P* = 0.02; FDR = 0.06). This was also significant using all TCGA samples (*P* < 0.001; FDR = 0.002). UTUC samples with *ZFP36* gene family mutations harbored a higher mutational load as compared to others (*P* = 0.008; Fig. 2f). This observation was validated in the TCGA bladder cancer dataset (*P* = 0.0009; Fig. 2g).

Next, we then looked at the expression of *ZFP36L1* in the FANTOM5 dataset and Human Protein Atlas and discovered that the bladder urothelium is one of the tissues with the highest mRNA and protein expression levels, highly suggesting the functional significance of this gene (Additional file 2: Fig. S4a-b).

Finally, a pooled analysis of 562 patients in three bladder cohorts (TCGA, DFCI, and BGI) showed that bladder carcinomas with *ZFP36* gene family mutations were associated with better overall survival as compared to others (*P* = 0.01; Fig. 2h); we also observed a trend toward improved progression-free survival in these patients (*P* = 0.08; Fig. 2i).

### Functional analysis of *ZFP36L1* knockdown

To investigate the biological role of *ZFP36L1*, we performed loss-of-function experiments of *ZFP36L1* using siRNA in the TCCSUP bladder cancer cell line (Additional file 2: Fig. S5a). Light microscopy images revealed disruption of the cell to cell junctions and clear change to spindle-shaped morphology in the cells with *ZFP36L1* knockdown (Additional file 2: Fig. S5b). This was associated with loss of E-cadherin expression, consistent with the epithelial-mesenchymal transition (EMT) (Additional file 2: Fig. S5a). While no effect of the *ZFP36L1* knockdown was observed regarding cell proliferation and apoptosis (Additional file 2: Fig. S5c-d), transwell assay showed a significant increase in cell migration in *ZFP36L1*-depleted cells as compared to control cells (Additional file 2: Fig. S5e).

### Mutational signature profiles of UTUC

**Fig. 3:**
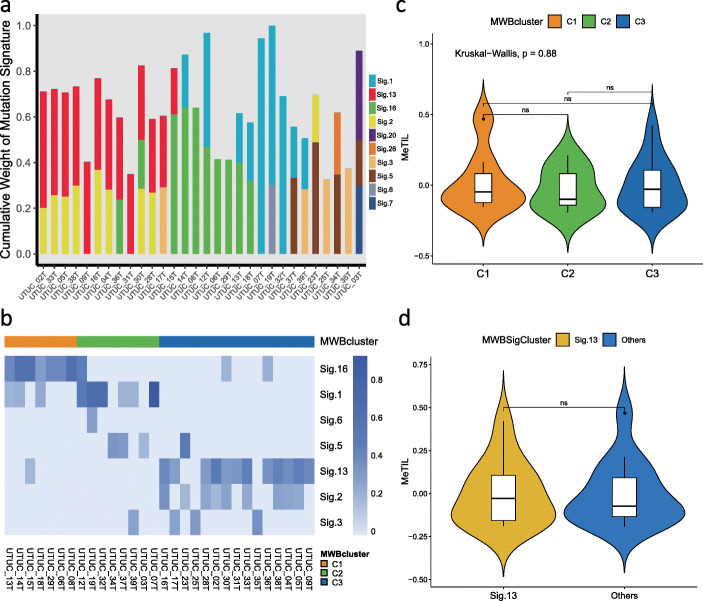
Analysis of mutational signatures and correlation with immunity. **a** Mutation signature analysis of upper tract urothelial carcinomas showing that signatures 1, 13, and 16 have the highest cumulative weight among the 30 signatures identified by Alexandrov. **b** Non-negative matrix factorization (NMF) showing three robust clusters based on the mutation weight matrix of different mutational signatures. MWBcluster C1 (*n* = 7), C2 (*n* = 8), and C3 (*n* = 15) were characterized by signature 16, signature 1, and signatures 13, respectively. **c** Violin plot showing no difference of DNA methylation tumor-infiltrating lymphocyte (MeTIL) score between the identified three clusters. **d** Violin plot showing no difference of DNA methylation tumor-infiltrating lymphocyte (MeTIL) score between signature 13 and other signatures. MWBcluter: mutation weight-based cluster

We then sought to decipher the heterogeneity of UTUC by characterizing the mutational signatures [10]. We found high variability between samples with signatures 1, 13, and 16 being the predominant ones (Fig. 3a). We applied a non-negative matrix factorization (NMF) that identified three robust mutation weight (MW)-based clusters considering different mutational signatures and determined signature contributors to each cluster (Fig. 3b). MWBcluster C1 (*n* = 7) was characterized by signature 16, of which etiology remains unknown. C2 (*n* = 8) was characterized by enrichment of signature 1, linked to the endogenous mutational process initiated by spontaneous deamination of 5-methylcytosine. Finally, C3 was enriched (*n* = 15) by the presence of mutational signatures 13 and 2, related to the activity of apolipoprotein B mRNA editing enzyme, catalytic polypeptide-like (APOBEC). As mutation signatures of APOBEC cytidine deaminase were associated with high rates of somatic mutations, and likely increased tumor-infiltrating lymphocytes (TILs), we asked whether either these three clusters or signature 13 was associated with TILs, inferred from DNA methylation (MeTIL) analysis for samples for which DNA methylation and WES were available. We did not find any association (Fig. 3c, d). Notably, UTUC tumors with *FGFR3* mutation showed a significantly lower MeTIL score as compared to others (*P* = 7.8 × 10^−5^; Additional file 2: Fig. S6a). This was also validated in the TCGA bladder carcinoma cohort (*P* = 9.0 × 10^−6^; Additional file 2: Fig. S6b). The immune infiltration derived by *FGFR3* mutation was independent from muscle invasiveness status (*P* = 0.008 for MI; *P* = 0.024 for NMI). We also found that UTUC MI samples tended to present with a higher MeTIL score relative to NMI ones (*P* = 0.089; Additional file 2: Fig. S6c).

### Detection of fusion transcripts and unsupervised clustering of gene expression

**Fig. 4:**
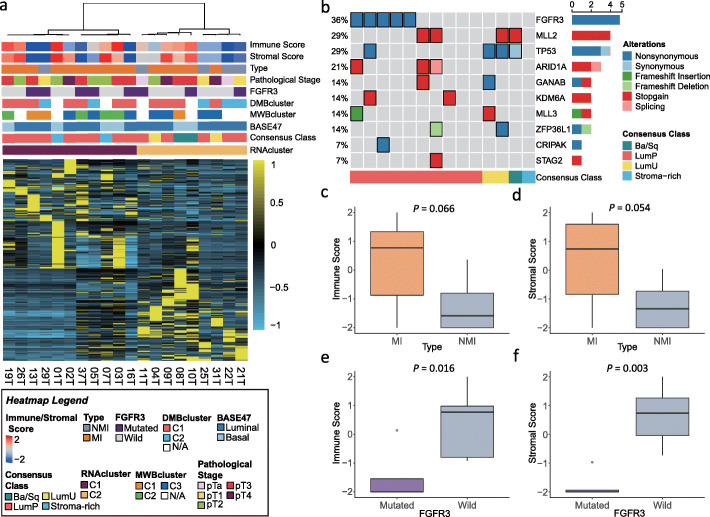
Analysis of upper tract urothelial carcinomas (UTUC) transcriptome. **a** Unsupervised mRNA clustering of 20 UTUC cases showing two clusters, with no clear association identified between them and clinical tumor features. **b** OncoPrint depicting UTUC subtypes according to the molecular consensus classification of muscle-invasive bladder cancers. Recurrent somatic mutations in those UTUC cases with available genetic data are also shown. **c**, **d** Immune and stromal gene expression scores in muscle-invasive (MI) and non-muscle-invasive (NMI) UTUC samples. **e**, **f** Immune and stromal gene expression scores in *FGFR3*-mutated and *FGFR3* wild-type UTUC samples. DMBcluster: DNA methylation-based epi-cluster; MWBcluster: mutation weight-based cluster; RNAcluster: RNA expression-based cluster

We first analyzed 20 UTUC (15 MI and 5 NMI) for fusion transcripts and detected only one case (UTUC-11) with the oncogenic *FGFR3-TACC3* fusion. No other fusions were identified. Unsupervised consensus hierarchical clustering identified two clusters (Fig. 4a). There was no difference observed between C1 and C2 regarding clinicopathological tumor features, probably due to the small cohort size (Additional file 1: Table S7). To better understand the biology of UTUC relative to bladder carcinomas, we applied the recently identified consensus molecular classification of muscle-invasive bladder carcinomas to our cohort. Fourteen cases (70%) were classified as luminal papillary, including all NMI cases (Additional file 1: Table S8). For the remaining cases, two were classified as luminal unstable, two as stromal-rich, and two as basal/squamous. None of the stromal enrich or basal squamous harbored *FGFR3* mutations (Fig. 4b). We also performed clustering of the gene expression using the BASE47 signature. We observed that the majority of our cases were “luminal-like” (*n* = 14), while 6 cases were classified as “basal-like” (Fig. 4a, Additional file 2: Fig. S7). Although the number was small, MI tumors tended to show higher enrichment for immune and stromal scores as compared to NMI tumors (*P* = 0.066 for immune scores; *P* = 0.054 for stromal scores; Fig. 4c, d). *FGFR3* mutations and/or fusions were associated with significantly lower immune and stromal scores (*P* = 0.016 for immune scores; *P* = 0.003 for stromal scores; Fig. 4e, f).

### DNA methylation subtype classification

**Fig. 5:**
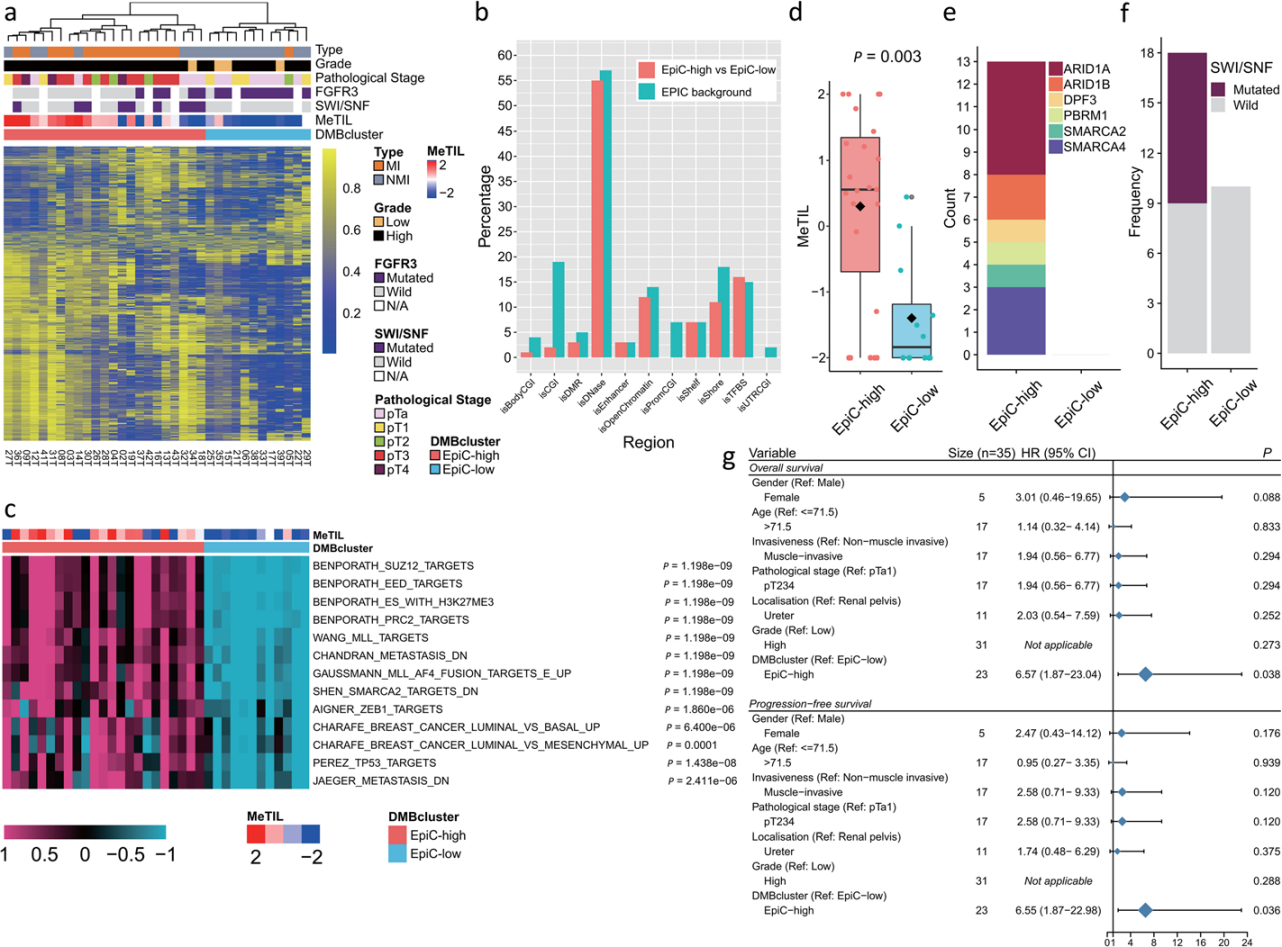
Analysis of upper tract urothelial carcinomas (UTUC) DNA methylation. **a** Unsupervised clustering of most variable DNA methylation probes in UTUC showing two epi-clusters: EpiC-C1(high) and EpiC-C2(low). **b** Percentage and distribution of differentially DNA methylation probes (blue) between EpiC-high and EpiC-low subgroups across different genomic regions annotated by EPIC arrays (red). Note that there is no enrichment identified in specific genomic regions. **c** Single-sample Gene Set Enrichment Analysis (ssGSEA) showing pathways enriched in EpiC-high relative to EpiC-low cluster. **d** Boxplot showing significantly higher MeTIL score in EpiC-high subgroup than that in EpiC-low subgroup (*P* = 0.003). **e** Count of mutations in SWI/SNF family genes in EpiC-high and EpiC-low clusters. **f** Frequency of mutations affecting SWI/SNF family genes in EpiC-high versus EpiC-low UTUC subgroups. Note that 9 out of 18 (50%) UTUC samples in the EpiC-high subgroup harbored mutations as compared to none in the EpiC-low subgroup. **g** Forest plot showing that the DMBcluster of EpiC-high and EpiC-low is a significant prognostic factor as compared to other major clinicopathological features for either overall survival or progression-free survival

To obtain subtype classifications of UTUC samples (*n* = 35), we performed unsupervised hierarchical clustering using the 1% most variable probes, after excluding probes with no available values and those located on sex chromosomes (*n* = 836,691). We identified two robust DNA methylation-based (DMB) epi-clusters: EpiC-C1 (*n* = 23; 65.7%) and EpiC-C2 (*n* = 12; 34.3%) (Fig. 5a). Heterogeneity within the two clusters is also supported by principal component analysis (PCA) (Additional file 2: Fig. S8). We did not observe enrichment for specific regions in the genome, which were more susceptible to DNA methylation changes (Fig. 5b). Notably, those occurred mainly outside the promoter CpG islands (Fig. 5b). To determine the differentially methylated probes (DMPs) and differentially methylated regions (DMRs) between the two identified epi-clusters, we harnessed the chip analysis methylation pipeline (ChAMP) using default parameters of EPIC arrays. Using the most stringent criteria, we picked up 14,243 significantly hypermethylated probes from a total of 242,687 DMPs. Among these, EpiC-C1 presented frequent hypermethylation, since a total of 14,209 probes were significantly hypermethylated as compared to EpiC-C2, whereas only 34 probes gained methylation in EpiC-C2. In this manner, we re-designated EpiC-C1 as EpiC-high and EpiC-C2 as EpiC-low accordingly. GSEA demonstrated that DMRs were enriched for pathways related to the polycomb repressive complex 2 (PRC2) and MLL targets. Interestingly, we also observed enrichment for SMARCA2 target genes and ZEB1 targets (Fig. 5c). Notably, the EpiC-low UTUC subtype harbored a significantly lower MeTIL score as compared to the MeTIL high subtype (*P* = 0.003) (Fig. 5d).

### Association between DNA methylation classification, genetic and clinicopathological tumor, and patient features

Integrative analysis of EpiC-low and EpiC-high UTUC samples with matched genetic landscape showed that EpiC-high UTUC samples were highly enriched for mutations in *SWI/SNF* genes (50% vs 0%, *P* = 0.01, FDR = 0.054; Fig. 5e, f), while samples classified as EpiC-low were enriched for *FGFR3* mutations (90% vs 28%, *P* = 0.004, FDR = 0.044; Fig. 5a, Additional file 1: Table S9).

In addition, EpiC-high UTUC samples were enriched for MI tumors (*n* = 16/23, 69.6%) in contrast to EpiC-low UTUC samples, which were enriched for NMI tumors (11/12, 91.7%, *P* = 0.0009; Additional file 1: Table S10). No other differences according to age and gender were found (Additional file 1: Table S10). We then analyzed the associations between clinicopathological tumor features, UTUC DMB epi-clusters, and patient survival (Fig. 5g). We found that patients with tumors belonging to the EpiC-high cluster had shorter overall survival as compared to those belonging to the EpiC-low subtype (*P* = 0.038; HR = 6.57; 95% CI 1.87–23.04; Additional file 2: Fig. S9a). Likewise, they had shorter distant metastasis-free survival as compared to those belonging to the EpiC-low subtype (*P* = 0.036; HR = 6.55; 95% CI 1.87–22.98; Additional file 2: Fig. S9b). No association with survival was identified for other clinicopathological tumor variables, which highlights the prognostic value of epi-clusters in UTUC.

### Hypomethylation of *FGFR3*-mutated UTUC

We then decided to assess how the epigenetic landscape of EpiC-high and EpiC-low tumors diverge from the normal urothelium. To do so, we performed unsupervised hierarchical clustering of UTUC (*n* = 35) and adjacent normal samples (*n* = 8); as expected, normal samples gathered together as compared to those from UTUC, which clustered into two groups (Additional file 2: Fig. S11). One UTUC cluster was *FGFR3*-enriched (*n* = 15/24) and the other was *FGFR3* wild-type (*n* = 0/11) (*P* = 0.0008), consistent with the notion that *FGFR3* mutation might be associated with distinct epigenome alterations. In this context, we looked for differentially methylated probes between *FGFR3*-mutated and wild-type UTUC samples. Overall, 84,717 (10.1%) out of 836,691 EPIC probes were differentially methylated (Δ*β* value ≥ 0.2 or ≤ − 0.2, FDR < 0.05), the majority being hypomethylated in *FGFR3*-mutated tumors (*n* = 82,991; 97.8%). The hypomethylated probes were mildly enriched in enhancers (3.8% vs 3.2%) (*P* < 0.001) and DNAse hypersensitive sites (63.7% vs 57.4%) (*P* < 0.001) (Additional file 2: Fig. S11a); in addition, those probes were overall related to 8136 differentially methylated regions (DMRs) (Additional file 1: Table S11). Among the top DMRs, we note many overlapping with genes known to be involved in bladder cancer, such as *FGFR3*, *GATA3*, *KRT15*, and *KRT5* (Additional file 2: Fig. S11b). GSEA found that those DMR were enriched for polycomb targets, as well as for several *FGFR* signaling pathways (Additional file 2: Fig. S11b).

### Subtype classification of bladder cancer cell lines according to DNA methylation

**Fig. 6:**
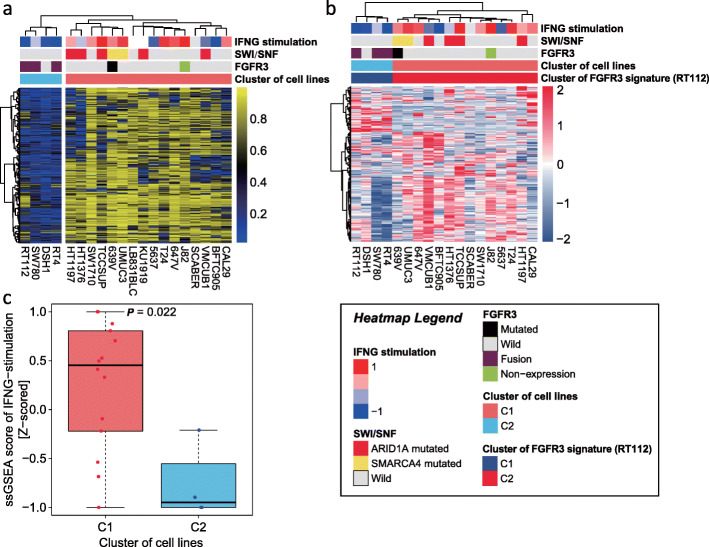
**a** Supervised clustering using hypermethylated probes derived from UTUC EpiC-high vs EpiC-low clusters and showing two subgroups of bladder cancer cell lines linked with SWI/SNF mutations (C1) and *FGFR3* translocation status (C2). Note that the probes depicted in the heatmap were those that were statistically significant. **b** Supervised clustering extracted from Mahe of bladder cancer cell lines according to *FGFR3* gene expression signature (using RT112 bladder cancer cell line). Note that although the DSH1 cell line does not harbor any *FGFR3* mutation, it clusters with the three other cell lines harboring *FGFR3* fusions and shares a similar transcriptomic program. **c** Box plot for interferon-γ stimulation signature score using single-sample gene set enrichment analysis (ssGSEA) in C1 and C2 clusters

To answer the question whether *FGFR3* somatic mutations can modulate DNA methylation in urothelial carcinomas, we thus decided to analyze the DNA methylation landscape of 20 bladder cancer cell lines. Notably, supervised clustering using the 14,209 hypermethylated probes derived from UTUC EpiC-high vs EpiC-low identified two subgroups tightly associated with *FGFR3* and *SWI/SNF* gene mutations (Fig. 6a). Notably, all but one cell line (DSH1) in the C2 (75%) cluster harbored *FGFR3* fusions with no *SWI/SNF* gene mutations. Conversely, only 2 out of 16 cell lines (12.5%) in the C1 cluster had *FGFR3* mutations; interestingly, *FGFR3* mutation is non-functional in J82 cell lines (no expression of *FGFR3*) and co-occurred with *SMARCA4* mutation in the 639V cell line. Supervised clustering of *FGFR3* gene expression signature showed similar findings (expression data was available for 18 cases) (Fig. 6b). Notably, expression of interferon-γ stimulation signature was upregulated in the C1 as compared to the C2 clusters (*P* = 0.022).

### Methylation of promoter genes in UTUC

We then looked at promoter genes located in the CpG islands, which were methylated in UTUC tumor samples with a frequency higher than 10%. We identified 905 genes methylated in our cohort (Additional file 1: Table S12). Notably, *ACTL6B*, a member of SWI/SNF, was frequently methylated (20% in tumors vs 0% in normal samples). Functional annotation analysis using DAVID identified that those are enriched for homeobox genes (*P* = 3.2 × 10^−40^), embryonic morphogenesis (*P* = 3.9 × 10^−13^), mesenchymal cell development (*P* = 1.8 × 10^−6^), and urogenital system development (*P* = 1.1 × 10^−4^). We then looked if there were any genes differentially methylated between MI and NMI tumors from one side, as well as *FGFR3*- versus non-*FGFR3*-mutated tumors from the other side. No statistically significant enriched promoter was found, suggesting that the majority of changes between *FGFR3*-mutated and *FGFR3* wild-type *FGFR3* tumors, as well as between MI and NMI tumors, arose outside promoters. No promoter methylation of *ZFP36L1* was identified in our cohort.

### Association of epigenetic UTUC two subtypes’ signatures with TCGA bladder muscle-invasive cancer cohort

**Fig. 7:**
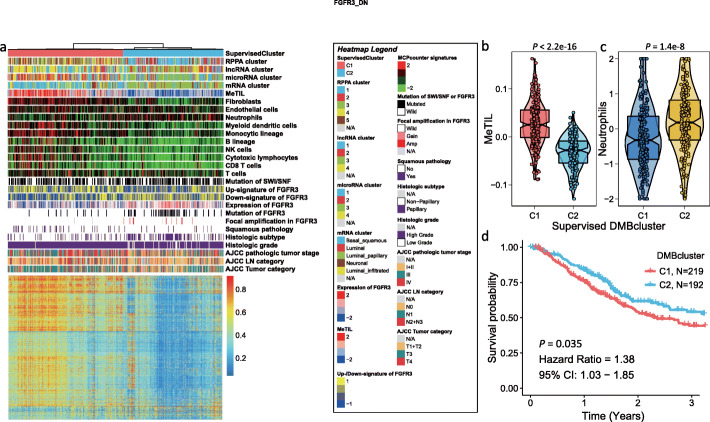
**a** Supervised consensus clustering of TCGA bladder cancers using hypermethylated probes derived from UTUC EpiC-high vs EpiC-low. Note the presence of two subgroups: BLCA-C1 and BLCA-C2. While the earlier is hypermethylated and immune enriched, the latter is enriched for *FGFR* mutations and *FGFR* amplification and displayed *FGFR3* overexpression. In addition, BLCA-C2 is an immune desert with no infiltration by T cells and fibroblasts. **b** Box plot showing the MeTIL score distribution according to EpiC signature. Note that BLCA-C1 harbored a higher score than the BLCA-C2 subgroup. **c** Box plot showing the neutrophils score distribution according to EpiC signature. Note that BLCA-C2 harbored a higher score than the BLCA-C1 subgroup. **d** Kaplan-Meier curves for the overall survival in the TCGA-BLCA cohort showing that the BLCA-C1 subgroup harbored a poor outcome as compared to the BLCA-C2 subgroup

We further decided to apply the 14,209 hypermethylated probes derived from UTUC EpiC-high vs EpiC-low to TCGA muscle-invasive bladder cancer cohort, where we identified through supervised consensus clustering two subgroups (Fig. 7a). Similar to the UTUC cohort, the BLCA-C1 presented a dramatically high methylation level and a higher MeTIL score as compared to BLCA-C2 (*P* < 2.2 × 10^−16^; Fig. 7b). While BLCA-C2 was enriched for tumors with low grade, low stage, and papillary differentiation, BLCA-C1 was enriched for tumors with high grade, high stage, and non-papillary differentiation (Additional file 1: Table S12). Notably, our classification correlates with mRNA, miRNA, RPPA, and DNA methylation subtype TCGA classification (Additional file 1: Table S13). Additionally, BLCA-C2 was enriched for *FGFR3* mutations (Additional file 1: Table S14), along with focal *FGFR3* amplification and higher expression of *FGFR3* (Fig. 7a). Notably, BLCA-C1 presented a significantly higher MeTIL score (*P* < 2.2 × 10^−16^) as compared to the BLCA-C2 cluster (Fig. 7b). We thus decided to use MCP-counter to deconvolute populations of immune and stromal cells in both groups; we found enrichment of fibroblasts, myeloid dendritic cells, monocytic cells, B cells, T cells, and cytotoxic CD8 T cells in the BLCA-C1 cluster, while BLCA-C2 was significantly enriched for neutrophils (*P* = 1.4 × 10^−8^; Fig. 7b, c). Finally, patients with tumors belonging to the BLCA-C1 subgroup had a poor median OS as compared to those belonging to the BLCA-C2 subgroup (*P* = 0.035; HR = 1.38; 95% CI 1.03–1.85). The median OS for patients in BLCA-C1 was 2.4 years (95% CI 1.9–3.7) and 5.4 years (95% CI 2.5–8.7) for those in the BLCA-C2 subgroup (Fig. 7d).

### Integrative clustering based on multi-omics data

To understand the crosstalk between genetic and epigenetic profiling on the 28 UTUC samples for which WES and DNA methylation profiling were available, we performed integrative clustering of somatic copy number variation (SCNV), mutation, and methylation data using iClusterBayes. All mutations of *SWI/SNF* genes, including *ARID1A*, were combined for integrative clustering. Two iClusters (i.e., iCluster-high [*n* = 17] and iCluster-low [*n* = 9]) were identified and distinguished EpiC-high and EpiC-low clusters (*P* = 4.9 × 10^−5^). Four mutation contributors were identified with a posterior probability greater than 0.5 (i.e., *FGFR3* [0.99], *SWI/SNF* [0.63], *KMT2D* [0.63], and *TP53* [0.56]) (Additional file 2: Fig. S12a). Among which, *FGFR3* (*P* = 0.011, FDR = 0.047) and *KMT2D* (*P* = 0.028, FDR = 0.057) were significantly enriched in iCluster-low, whereas *SWI/SNF* (*P* = 0.098, FDR = 0.13) show a tendency for being enriched in iCluster-high (Additional file 1: Table S15). Additionally, 6971 methylation probes and four copy number alterations made contributions to the clustering process (Additional file 2: Fig. S12b-c). Copy number alteration contributors comprise deletion of both *1p36.33* (0.66) and *9p21.3* (0.54) and amplification of both *8p11.23* (0.63) and *8q22.3* (0.85), where amplification of *8q22.3* was significantly associated with iCluster-high (*P* = 0.023, FDR = 0.093) (Additional file 2: Fig. S12d, Additional file 1: Table S15). Notably, 8q22.3 contains genes for which amplification was recently shown to define aggressive bladder cancer [11].

## Discussion

We performed the first comprehensive genomic and epigenomic profiling of UTUC with the goal to identify molecular underpinnings of muscle invasiveness and determine novel key drivers linked with clinicopathological tumor features and patient outcome. We identified two UTUC methylome subtypes (i.e., EpiC-low and EpiC-high), which were enriched for NMI and MI samples, respectively. While the former one was hypomethylated, immune-depleted, and enriched for *FGFR3* mutations, the latter one was hypermethylated, immune-infiltrated, and tightly associated with somatic mutations of SWI/SNF genes. Notably, we observed similar findings in bladder carcinoma cell lines, suggesting that *FGFR3* alterations (i.e., fusions, mutations) might alter the methylome via direct or indirect mechanisms. This is also consistent with the notion that loss of function of SWI/SNF genes leads to the inability of the complex to counteract polycomb [12], which controls DNA methylation [13].

In addition to other mutations known to be altered in UTUC, we discovered for the first time a high rate of mutations of the *ZFP36L1* gene. The majority of those were truncating mutations and likely oncogenic. If we consider mutations affecting all *ZFP36* family genes, those were present in more than one quarter among UTUC cases. These results have not been identified previously in two different cohorts [4, 5]. This might be related to either the low number size of the cohorts analyzed or to the limited accurate detection in WES of InDels, often more challenging than SNVs [14]. Future studies are needed to clarify the true incidence of those mutations and their clinical relevance. As a zinc-finger RNA-binding protein regulating several cytoplasmic AU-rich elements (ARE), *ZFP36L1* ultimately attenuates protein synthesis through the degradation of several mRNA transcripts. Using loss-of-function experiments, we observed increased cell motility of a bladder cancer cell line, likely through EMT, although we did not detect any effect on cell proliferation and apoptosis. These data are consistent with recently reported functional analysis using in vitro and in vivo experiments, demonstrating that *ZFP36L1* suppress hypoxia and cell cycle signaling in bladder carcinomas [15]. Thus, *ZFP36L1* mutations might lead to an increase in cell migration, bolstering tumor progression. In addition, UTUC cases harboring *ZFP36* family gene mutations displayed higher tumor mutational loads, an observation which we validated in the TCGA bladder carcinoma dataset. These data are consistent with the notion that a higher tumor mutational load is positively associated with a better survival prognosis in numerous cancer types [16].

Previous studies investigated mutations of UTUC and identified a small proportion of cases with germline mutations in MMR genes, with the majority of tumors occurring sporadically [6]. Consistent with these recent findings, none of our sporadic UTUC has a mutator phenotype, and the majority of cases have luminal papillary-enriched transcriptome signatures, although caution might be considered in interpreting the results, as almost half of the cases assessed had *FGFR3* mutations and were NMI tumors.

Besides genetic alterations, we investigated for the first time, to our knowledge, DNA methylation landscapes of both muscle-invasive and non-muscle-invasive UTUCs using an epigenome-wide approach. We discovered that DNA methylation is capable of distinguishing two distinct epigenetic UTUC subtypes, linked with muscle invasiveness status. This classification might be used in the future, if validated, to predict outcomes of resected UTUC patients and help stratify those that may benefit from preoperative chemotherapy or adjuvant *FGFR3*-targeted agents [17]. The discovery of an *FGFR3*-mutated UTUC subtype which is hypomethylated and immune-depleted, as compared to *FGFR3* wild-type tumors, is novel. Future mechanistic data are needed to better understand if the epigenetic remodeling occurs directly or indirectly. Another observation that also deserves to be discussed is the link between immunity and SWI/SNF genetic tumor alterations which we showed to be associated with higher TILs in UTUC. In addition, bladder cell lines without *FGFR3* alterations and which harbored SWI/SNF gene mutations showed higher interferon-γ stimulation signature. However, whether this association could be explained mechanistically by SWI/SNF mutations is unclear. Further studies are needed to clarify these findings in UTUC, given that the link between mutations in SWI/SNF genes and immunity is contradictory in different cancer subtypes [18, 19].

In colorectal cancer, a positive correlation was found between oncogenic BRAF mutations and hypermethylation of multiple promoter CpG islands, known as CpG island methylator phenotype [20]. A high level of mutations of SWI/SNF genes in *FGFR3* wild-type UTUC tumors might explain their CpG island hypermethylation, as SWI/SNF genes have been shown to antagonize the polycomb repressive complex 2 (PRC2) [12].

From a therapeutic standpoint, our data suggest that EpiC-Low UTUC might benefit from the combination of *FGFR3* inhibitors with PD-1/PD-L1 inhibitors as a targeted therapeutic strategy to modulate the T cell-depleted phenotype, consistent with the results of a recent study [5]. Indeed, erdafitinib was granted accelerated approval by the FDA in relapsed/refractory metastatic bladder cancer on the basis of phase 2 trial results, showing a response rate of 40% in 90 patients with tumors that harbored actionable *FGFR* alterations [21]. For the remaining tumors with *SWI/SNF* mutations, EZH2 inhibition with and without cisplatin might represent an effective option, as it has been recently demonstrated in bladder cancer cells and xenografts, through a mechanism that activates a natural killer (NK) cell-based immune response [22].

Our study is the first to propose two distinct routes of UTUC carcinogenesis through a crosstalk between genetic and epigenetic alterations. We used multi-omics data of a very well-annotated monocentric cohort with comprehensive integration of a wealth of data. However, our study has some limitations, including a small number of cases analyzed due to the relative rarity of tumor type, although we have been able to validate some of our observations in cohorts of bladder carcinomas. Further confirmation of our findings in larger UTUC cohorts is warranted. Future studies also need to examine the stability of the molecular profiles we identified in UTUC across matched primary and metastatic tumors.

In summary, our findings define the foundation of the molecular basis of UTUC heterogeneity. We also provide a roadmap for the rational clinical development of targeted and immunotherapeutic strategies that are specific to UTUC, but also potentially applicable to other tumor types harboring *FGFR3*-activating molecular alterations.

### Experimental models and subjects details

#### Sample collection and histopathological analysis

Fresh-frozen UTUC of 40 cases was collected retrospectively from the Pitié-Salpetrière Hospital Biobank and re-evaluated histopathologically by one expert pathologist (E.C.). Overall, DNA and RNA of good quality and quantity were obtained for 40 and 20 cases, respectively. Detailed clinical and pathologic characteristics of the cohort are reported in Additional file 1: Table S1. Matched germline DNA from adjacent bladder tissues was also collected in 30 cases. Whole-exome sequencing (WES) was performed for 30-paired UTUCs and adjacent normal tissue. RNA sequencing was performed on 20 UTUC cases; in addition, DNA methylation was analyzed using Infinium EPIC arrays on 35 UTUC cases and 8 normal adjacent bladder tissues.

#### Ethical approval

All patients had previously provided written informed consent for tumor collection and subsequent analysis. The collection and use of tissues followed the procedures that are in accordance with the ethical standards formulated in the Declaration of Helsinki. The study has been approved by the ethical committee of the Pitié-Salpêtrière Hospital (IDF-6, Ile de France).

#### Nucleic acid extraction

DNA extraction was performed using the DNeasy Blood & Tissue Kit (Qiagen) according to the manufacturer’s instructions. RNA extraction was performed using the RNeasy Kit (QiagenQIAGEN) according to the manufacturer’s instructions. Quality control of extracted nucleic acids was done using an Agilent 2100 Bioanalyzer.

#### Whole-exome sequencing and somatic mutation detection

Exome capture was performed using Agilent SureSelect Human All Exon 50 Mb according to the manufacturer’s instructions. Briefly, 3 mg of DNA from each sample was used to prepare the sequencing library through shearing of the DNA, followed by ligation of sequencing adaptors. Whole-exome sequencing was performed, and paired-end sequencing (2 × 76 bp) was carried out using the Illumina HiSeq 2000; the resulting data were analyzed with the Illumina pipeline to generate raw FASTQ files. The coverage of our germline samples and tumor samples varied between 43–80× and 79–158×, respectively. The technical details and mutation detection were done according to the pipeline we previously reported [23]. We filtered out all known single-nucleotide variants (SNVs)/indels in the UCSC dbSNP 135 and 1000 Human Genome Project SNP databases, and kept any mutations, which are in the Catalogue of Somatic Mutations in Cancer (COSMIC) database, curated by the Wellcome Trust Sanger Institute. The variation classification for each mutation was annotated by ANNOVAR. Each somatic mutation or indel was annotated with its functional effect by SIFT to determine whether a mutation candidate was synonymous or nonsynonymous (benign or deleterious). Mutations that failed to be annotated by ANNOVAR, labeled with “unknown,” were removed first for downstream analysis. We used MutSigCV_v1.41 (www.broadinstitute.org) to infer the most significantly mutated genes across these samples, and 10 frequently mutated (> 10%) genes that past test of significance were identified (*P* < 0.05) [24]. The mutation landscape for those 10 significantly mutated genes across 30 UTUC samples was shown by OncoPrint using R package *ComplexHeatmap* [25]. The tumor mutational load per megabase was computed by summing all types of mutations, divided by 50 MB. We harnessed mutation signatures to decipher samples that shared similar mutational spectra. All non-single-base substitutions (e.g., insertions, deletions, and complex multi-base substitutions) were filtered out of the table, leaving single-base substitution mutations annotated as nonsense, missense, or coding silent substitutions [26]. Mutational signatures described by Alexandrov et al. [10] and curated at http://cancer.sanger.ac.uk/cosmic/signatures were evaluated using R package *deconstructSigs* [27] with the following parameters: “exome2genome” trinu-cleotide-count normalization and signature cutoff at 0.2. Unknown signatures were subsequently discarded. We used non-negative matrix factorization (NMF) by R package *NMF* with the method of “lee” and rank number of three to deconvolute the mutational signature landscape and determine the signature contributor for each class where contributors were identified by extractFeatures() with “max” method [28, 29].

#### Validation of somatic mutations by Sanger sequencing

Validation of selected somatic variants in three genes, including *FGFR3*, *KDM6A*, and *ZFP36L1*, was performed on DNA extracted from UTUC and normal adjacent samples analyzed by WES. In addition, *FGFR3* hotspot mutations (S249C and Y373C) were investigated in the remaining samples not analyzed by WES (*n* = 5). The genomic region surrounding the putative mutations was amplified with polymerase chain reaction (PCR) using specific primer pairs designed with the Primer Express 3.0 Software (Applied Biosystem). PCR products were then purified with the Qiaquick PCR purification kit (Qiagen, Milan, Italy) and sequenced on both strands using the Big Dye Terminator v1.1 Cycle Sequencing kit (Applied Biosystems). Sanger sequencing was performed on ABI 3730 Genetic Analyzer (Applied Biosystems).

#### RNA sequencing

Total RNA for 20 UTUC samples was converted into a library of template molecules for sequencing on the Illumina HiSeq 2000 according to the NuGen Ovation RNA-Seq System V2 protocol. In brief, first, single-stranded cDNA was synthesized from 100 ng of DNase1-treated total RNA using a mix of DNA/RNA chimeric primers that hybridize to both the 50 portions of the poly (A) sequence and randomly across the transcript. Second, strand synthesis produced double-stranded cDNA, which was amplified using single-primer isothermal strand displacement amplification. The resultant cDNA was fragmented to 200 bp (mean fragment size) with the S220 Focused-ultrasonicator (Covaris) and used to make barcoded sequencing libraries on the SPRI-TE Nucleic Acid Extractor (Beckman-Coulter). Libraries were quantitated by qPCR (KAPA Systems), multiplexed, and sequenced, four samples per lane, on the HiSeq2000 using 75-bp paired-end sequencing. The resulting data were analyzed with the current Illumina pipeline to generate raw FASTQ files. The raw, paired-end reads were aligned to the human reference genome, GRCh37/hg19, using the MOSAIK alignment software. MOSAIK works with paired-end reads from Illumina HiSeq 2000 and uses both a hashing scheme and the Smith-Waterman algorithm to produce gapped optimal alignments and to map exon junction-spanning reads with a local alignment option for RNA-seq. The resulting alignments were then saved as a standard bam file. We then counted the mapped reads in mRNA annotated in GENCODE15 to generate the raw counts for each gene using the HTSeq-count script distributed with the HTSeq package. We calculated the number of fragments per kilobase of non-overlapped exon per million fragments mapped (FPKM) [30]. To reduce noise, we filtered out low expressed mRNA, if its FPKM value is lower than 1 in at least 90% of the samples.

#### Unsupervised clustering for UTUC mRNA profile

Implementation of FPKM transformation and the filter procedure resulted in 12,492 unique genes with reliably measured expression. The gene expression data were then median centered in both directions and log_2_ transformed prior to clustering. Then, we performed unsupervised hierarchical clustering with *k* = 2 as the number of clusters by basically using the hclust() R function with Ward’s clustering method and Manhattan distance measures for each run. The consensus process was set to 80% of features and samples re-sampling with 500 perturbations. The final hierarchical clustering based on the consensus matrix used a distance measurement of Manhattan with Ward’s clustering method. Differential expression analysis was executed by the use of the *edgeR* R package, fitting a negative binomial generalized log-linear model [31].

#### DNA methylation and bioinformatics analysis

Global DNA methylation was assessed using the Infinium HumanMethylation850 (EPIC) BeadChip Array. Briefly, genomic DNA (500–1000 ng) was bisulfite-converted using the Zymo EZ DNA methylation kit (Zymo Research, Irvine, CA) according to the manufacturer’s recommendations. The amount of bisulfite-converted DNA and the completeness of the bisulfite conversion for each sample were assessed using a panel of MethyLight-based real-time PCR quality control assays. Bisulfite-converted DNA was then used as a substrate for the Illumina EPIC BeadArrays, as recommended by the manufacturer. Specifically, each sample was whole-genome amplified (WGA) and then enzymatically fragmented. Samples were then hybridized overnight to an 8-sample BeadArray, in which the WGA-DNA molecules annealed to locus-specific DNA oligomers linked to individual bead types. After the chemical processes, BeadArrays were scanned and the raw signal intensities were extracted from the *.IDAT files using the “noob” function in the minfi R package. The “noob” function corrects for background fluorescence intensities and red-green dye bias. The beta (*β*) value for each probe was calculated as M/(M + U), in which *M* and *U* respectively refer to the (pre-processed) mean methylated and unmethylated probe signal intensities. Probes with measurements in which the fluorescent intensity was not statistically significantly above the background signal (detection *P* value > 0.05) were removed from the dataset.

Pattern discovery of the methylation EPIC profile was performed in 35 UTUC samples. Any methylation probe that was located in a sex chromosome or that had at least one NA value was removed out of the total 866,091 probes. In those filtered (836,691), we picked the top 1% highly variable DNA methylation probes and performed unsupervised hierarchical clustering using the Euclidean distance and Ward’s clustering methods. Clusters (*k* = 2) were generated to methylation C1 and C2 based on the cutree() function. Differentially methylated probes (DMPs) and differentially methylated regions (DMRs) were achieved through the standard process of *ChAMP* with arraytype of EPIC [32]. To be specific, we adjusted the *P* value threshold for DMP detection, set to 0.05, and used the Benjamin-Hochberg and DMRcate methods to define DMRs. Let us denote $\overline{\beta_k^{C1}}$ as the mean *β* value of probe *k* in C1 and $\overline{\beta_k^{C2}}$ as that in C2. We determined probe *k* as the significantly hypermethylated probe in C1 if $\overline{\beta_k^{C1}}\ge 0.4$, $\overline{\beta_k^{C2}}\le 0.2$, and FDR < 0.05. Using the above threshold, we identified 14,209 significantly hypermethylated probes in C1.

#### Data acquisition of external BLCA and UTUC cohorts

We extracted three omics data from the TCGA-BLCA cohort where gene expression and DNA methylation data were downloaded from UCSC Xena (https://xena.ucsc.edu/), and somatic mutation data and detailed clinicopathological information were obtained from cBioPortal (http://www.cbioportal.org/) under the archive of Bladder Cancer [33]. Overall, 412 muscle-invasive bladder carcinoma samples were used for DNA methylation and somatic mutation analysis, and 407 samples for expression data. Second, we included the Hurst cohort [9] which contains somatic SNVs and small insertions/deletions identified by whole-exome sequencing for 24 TaG2 bladder tumors. Third, we collected omics data from urinary tract cell lines. Corresponding DNA methylation profiling for 20 cell lines and expression array data for 18 cell lines (2 out of 20 have no data for expression) were retrieved from Genomics of Drug Sensitivity in Cancer (GDSC, https://www.cancerrxgene.org/). Specific mutation of *FGFR3* and SWI/SNF pathway was assessed from cBioPortal (https://www.cbioportal.org/) under the archive of Cancer Cell Line Encyclopedia (Broad 2019). Only SWI/SNF gene mutations considered as oncogenic drivers were included. *FGFR3* signature was collected from differential expression analysis of RT112 cell line treated with FGFR3 siRNA according to the literature [34]. Interferon-γ stimulation signature was retrieved from a previous study [35].

#### Subtype inference for UTUC and BLCA tumors

We used R package *consensusMIBC* [36], which implements a nearest centroid transcriptomic classifier that assigned class labels according to the consensus molecular classification of MIBC to infer consensus subtype of UTUC and TCGA-BLCA. Use function of getConsensusClass() with normalized expression data and parameters by default, one of the following six molecular classes will be assigned due to the highest correlation coefficient, including luminal papillary (LumP), luminal non-specified (LumNS), luminal unstable (LumU), stroma-rich, basal/squamous (Ba/Sq), and neuroendocrine-like (NE-like). We also inferred the BASE47 subtypes for our UTUCs based on a 47-gene signature according to the literature [37] by using unsupervised hierarchical clustering with distance measurement of 1-Pearson’s coefficient and linkage function of Ward.D2.

#### Supervised clustering of BLCA methylation 450k profile

BLCA methylation data contains DNA methylation *β* value of 433 BLCA samples with 412 tumor samples and 21 normal samples assessed by TCGA using the Illumina Infinium HumanMethylation450 platform. Out of 14,209 hypermethylated probes derived from UTUC EpiC-high vs EpiC-low, a total of intersected 3790 hypermethylated probes was used for supervised clustering of the TCGA-BLCA methylation 450k profile. To assess the stability of the discovered clusters, we performed a consensus hierarchical clustering. We conducted 500 runs of hierarchical clustering on the resampled data. For each run, 80% samples and 80% features were randomly chosen. The distance measurement was set as Manhattan, and the linkage function was set as Ward.D2. Based on the 500 runs, a consensus was obtained by taking the average over the connectivity matrices of every perturbed dataset. Then, we carried out hierarchical clustering with the consensus matrix as a similarity matrix, with a distance measurement of 1-Pearson’s coefficient and linkage function of Ward.D2.

#### Detection of frequently methylated genes

For genes having more than one probe mapping to its promoter, the median *β* value was considered to get 12,066 methylation genes. To minimize the influence of normal tissue contamination in DNA methylation data, we excluded methylation genes found in more than 50% normal samples with a *β* value ≥ 0.2 or genes with a median *β* value ≥ 0.2 in normal samples, and 11,346 methylation genes remained. We determined a gene’s methylation status by a *β* value cutoff of 0.3 and calculated its methylation percentage across all UTUC samples. A frequently methylated gene was defined if the corresponding percentage was greater than 10%.

#### Gene set enrichment analysis

For gene set enrichment analysis based on gene expression data, R package *clusterProfiler* was used for the pre-ranked gene list (descending ordered log2FoldChange value) derived from differential expression analysis [38, 39]. Molecular Signature Database gene sets were tested by using the gene set of msigdb.v6.0.symbols.gmt downloaded from the GSEA website (http://software.broadinstitute.org/gsea/). GSEA for DNA methylation were executed by the embedded champ.GSEA() function in ChAMP with the typical Fisher’s method. The enrichment scores of molecular pathways were evaluated using the gene set variation analysis method via R package *GSVA* [40, 41].

#### Integrative analysis of genetic and epigenetic profiling

Bayesian integrative clustering was performed by R package *iClusterPlus* by using the iCluster Bayesian method [42]. We applied this method on three available data types: genetic mutation, DNA methylation, and copy number alteration data with 26 shared samples. Basically, the mutation data contains 10 significantly mutated genes that were revealed by the MutSigCV algorithm with a *P* value of less than 0.05 and a mutational frequency greater than three cases. We further curated the mutation data by removing *ARID1A* but attaching SWI/SNF pathway mutation. We used the top 1% most variable probes as the methylation dataset. An under a confidence level of 0.75, amplification or deletion of arm with a *q* value less than 0.25 were selected, including 7 amplification peaks and 3 deletion peaks. For the sake of integrative analysis, the copy number was transformed into a binary term (e.g., 1 for deletion or amplification and 0 for no change). The parameter list was set as *K* = 1 (two clusters), n.burnin = 18,000, n.draw = 12,000, prior.gamma = 0.5 for the indicator variable gamma of each data set, sdev = 0.05, and thin = 3. We fitted binomial distribution for mutation data and copy number data and Gaussian for methylation data. The clustering contributor was considered if its corresponding posterior probability was greater than 0.5.

#### Copy number variation analysis

Recurrent focal somatic copy number alterations were detected and localized using GISTIC2.0 [43, 44] with the thresholds of copy number amplifications/deletions being equal to ± 0.15 and *q* value threshold being equal to 0.2.

#### Quantify the immune and stromal level of UTUC samples

The population abundance of tissue-infiltrating immune and stromal cell populations was estimated by R package *MCPcounter* per sample using normalized count data [45]. The presence of infiltrating immune/stromal cells in tumor tissue was estimated by R package *ESTIMATE* [46]. DNA methylation-based immune infiltration scores (MBII scores) were extracted by TCGA previous work [47]. Additionally, the individual DNA methylation of tumor-infiltrating lymphocyte (MeTIL) score was calculated according to the literature [48].

##### Cell line culture

TCCSUP cell line was purchased from ATCC and authenticated. Cells were tested and were verified for free of *Mycoplasma* contamination. These cells were grown in (EAGLE, 10% FCS, 0.1 mM AANE, 1 mM sodium pyruvate, 40 μg/ml gentamicin) media.

##### ZFP36L1 knockdown

3 × 10^5^ cells were plated in each well of a 6-well plate. After 24 h, cells were transfected with selected siRNA. For transfection, 4 μl of 5 nM of Human *ZFP36L1* (#L-011816-00-0005) siRNA smart pool or non-silencing pool siRNA (Cont siRNA) (#L-011816-00-0005) (Dharmacon) was diluted in 100 μl of OptiMEM media. In a separate tube, 8 μl of Lipofectamine RNAi Max reagent (# 2185383, Invitrogen™) was diluted in 100 μl of OptiMEM media. After 5 min of incubation, both the solutions were mixed and kept for 15 min incubation at room temperature. Later, the mixture was added drop wide to each well. The pictures of cells after 48 h from transfection were taken with macro-microscope with × 10 magnification. Later, the transfected cells were used for the Western blots and functional assays.

##### Western blots

After 48 h of transfection, the cells were scrapped from the plate in a tube and washed with PBS (1×). After washing, the cells were centrifuged, and the supernatant was discarded. The pellet was suspended in twice the volume of the LSDB buffer and 1× protease inhibitor (# 40091500, Sigma-Aldrich) and kept in liquid nitrogen for 2 min. Later, the sample was transferred in a water bath at 37 °C for 2 min. The incubation steps were repeated twice, and then the sample was centrifuged at 4 °C. After centrifugation, the supernatant was transferred to another tube. The total protein concentration was determined by dissolving 1 μl of total protein extract in 1× protein assay dye (# 5000006, Bio-Rad) and quantifying the amount by the Bradford dye-binding method. The protein samples were loaded in NuPAGE (4–12% bis-tris Gel, # 20070610, Invitrogen™) for electrophoresis and later transferred on the PVDF. The membrane first was blocked with 5% milk and then probed with the primary antibody overnight. The next day, the membrane was washed with PBST (1× PBS, 0.01% Tween 20) and then blocked with the secondary antibody. After incubation, the membrane was washed with the PBST, and full blots were treated with ECL (# UC180107, Protein biology) for the acquisition of the signal using the imager system. The Primary antibody used were ZFP36L1 (1:1000; #2119, Cell Signaling Technology), E-cadherin (1:1000; #9782, Cell Signaling Technology), and actin (1:5000).

##### Cell proliferation

The cell proliferation was determined using Invitrogen™ CellTrace™ (#2161821) according to the manufacturer’s instructions. 3 × 10^5^ cells were plated in a 6-well plate. The next day, the cells were transfected with either si ZFP36L1 or si control, and after 7 h, they were treated with cell trace violet dye for 20 min. The dye was replaced with (EAGLE, 10% FCS, 0.1 mM AANE, 1 mM sodium pyruvate, 40 μg/ml gentamicin) media. After 72 h transfection, the cell was trypsinized and centrifuged. The pellet was dissolved in PBS, and cells were used to analyze the cell cycle distribution using flow cytometry (BD. FORTRESSA X20). The data obtained was analyzed using the FlowJo software. The numbers are expressed as mean ± SEM of the percentage of low-proliferating cells, and three independent experiments were performed.

##### Cell apoptosis

After 24 h from plating, 3 × 10^5^ cells were transfected with either the si *ZFP36L1* or si control. After 72 h transfection, the cells were trypsinized and centrifuged at 4 °C. The pellets were treated with 100 μl of BD cytofix/cytoplasm fixation permeabilization solution (Kit #0071517, BD. Biosciences) and kept in ice for 20 min. Later, the cells were washed with the washing buffer (1× BD wash buffer in 1% BSA solution) and centrifuged at 4 °C. The supernatant was discarded, and the pellet was treated with PE rabbit anti-active caspase-3 (# 55082, BD. Biosciences) for 30 min kept in dark at room temperature. The final washing was done with the washing buffer (1× BD wash buffer in 1% BSA solution) and centrifuged at 4 °C. The supernatant was discarded, and the pellet was dissolved in PBS and analyzed for cascade 3 activity using flow cytometry (BD. FORTRESSA X20). The data obtained was analyzed using the FlowJo software. The numbers are expressed as mean ± SEM of % of apoptotic cells, and three independent experiments were performed.

##### Transwell assay

The transfected TCCSUP cells with either si ZFP36L1 or si control after 72 h were used for the transwell assay. The transfected cells (5 × 10^4^) were taken in 200 μl (EAGLE, 0.01% FCS, 0.1 mM AANE, 1 mM sodium pyruvate, 40 μg/ml gentamicin) media and plated on top of the 8-μm Transwell filter membrane in a 24-well plate. Using a pipette, 600 μl (EAGLE, 10% FCS, 0.1 mM AANE, 1 mM sodium pyruvate, 40 μg/ml gentamicin) media was added in the lower chamber of the 24-well plate. After 72 h of incubation, the membranes were washed with PBS (1×) and were fixed in 3.4% formaldehyde by adding 600 μl in the lower chamber and 200 μl in the upper chamber. The membranes were washed with PBS (1×) and then incubated in DAPI (1 μg, 20 μl/ml) for 30 min. After incubation, the cells from the above chamber were removed carefully with the cotton bud and washed with PBS (1×). The membranes were later viewed under the inverted microscope (ZEISS observer), and pictures were taken to count the number of cells. The counts of migrated cells are expressed as mean ± SEM of at least three independent experiments, each performed in triplicate.

### Statistical analyses

All statistical tests were executed by R/3.6.2, including Fisher’s exact test for categorical data, a two-sample Mann-Whitney test for continuous data, a log-rank test Kaplan-Meier curve, and Cox proportional hazards regression for hazard ratio (HR) with 95% confidence interval (95% CI) [49, 50]. Survival analysis was performed by R package *survival*. Mutual exclusivity analysis was done using one-side Fisher’s exact test, where there was an alternative parameter of “less” for mutual exclusivity and “greater” for co-occurrence analysis. For unadjusted comparisons, a two-sided *P* < 0.05 was considered statistically significant.

## Supplementary Information

**Additional file 1: Table S1**. Clinicopathological tumor features of upper-tract urothelial carcinomas samples used for the study. **Table S2**. Association between clinical variables and patients progression-free and overall survival. **Table S3**. List of somatic mutations identified in the whole-exome sequencing of upper-tract urothelial carcinomas (*n* = 30). **Table S4**. List of significantly mutated genes identified by the MutSigCV algorithm. **Table S5**. Mutual exclusivity and co-occurrence analysis for the 10 most significantly mutated genes using the one-side Fisher’s exact test. **Table S6**. Association between top 10 frequently significantly mutated UTUC genes and muscle-invasive status. **Table S7**. Association between UTUC RNA unsupervised clustering and clinical variables. **Table S8**. Subtypes of UTUC samples using consensus molecular classification of muscle-invasive bladder cancer. **Table S9**. Association between top 10 frequently significantly mutated genes and DNA methylation-based clusters in UTUC samples. **Table S10**. Association between clinical variables and DNA methylation UTUC based clustering in samples with EpiC-low and EpiC-high signatures. **Table S11**. List of differentially methylated regions (DMR) using hypomethylated probes in *FGFR3*-mutated UTUC cases as compared to *FGFR*-wild type samples. **Table S12**. List of genes with promoter CpG islands methylation in UTUC tumor samples. Only those with a frequency higher than 10% are reported. **Table S13**. Association between UTUC EpiC signature and clinical variables in TCGA bladder carcinomas cohort. **Table S14**. Association between significant mutations of UTUC and supervised TCGA DNA methylation based clustering. **Table S15**. Association between mutation and copy number contributors and iClusters.

**Additional file 2: Fig. S1**. Copy number variants analysis. **Fig. S2**. Comparative analysis of the frequency of most frequent mutations in upper-tract urothelial carcinomas (UTUC) as compared to urothelial bladder carcinomas (UBC). **Fig. S3**. *ZFP36* family mutation in diverse histopathological cancer subtypes. **Fig. S4**. a) Human protein atlas and FANTOM5 datasets showing that protein expression and RNA expression of *ZFP36L1* is the highest and the most expressed in the urinary bladder as compared to other tissues. **Fig. S5**. *ZFP36L1* inhibition increases cell motility in vitro. **Fig. S6**. Distribution of MeTIL score between FGFR3-mutant and wild type of UTUC and UBC, and between muscle-invasive and non-muscle invasive UTUC samples. **Fig. S7**. Heatmap of BASE47 bladder signature in UTUC samples showing two subgroups of “luminal-like” and “basal-like”. **Fig. S8**. Principal component analysis showing heterogeneity of the two epi-clusters EpiC-C1 and EpiC-C2, obtained through unsupervised clustering of most variable DNA methylation probes. **Fig. S9**. Kaplan-Meier curves for progression-free survival regarding UTUC epi-clusters. **Fig. S10**. Unsupervised clustering of DNA methylation using most variable probes in the whole dataset encompassing UTUC (*n* = 35) samples and adjacent normal urothelium (*n* = 8). **Fig. S11**. Distribution of DNA methylation probes across different genomic regions significantly hypomethylated in *FGFR3*-mutated tumors versus *FGFR3*-wild type as compared to EPIC arrays. Heatmap showing differentially methylated probes located in top ranked DMR and corresponding ssGSEA results. **Fig. S12**. Integrative clustering analysis of multi-omics data. Full Western blots containing the entire ladder for loss-of-function experiments of *ZFP36L1* using siRNA in TCCSUP bladder cancer cell line.

**Additional file 3.** Review history (LOG 180 bytes).

## Data Availability

RNA sequencing data generated in this paper have been deposited at the NCBI Sequence Read Archive (SRA) hosted by the NIH (SRA accession: PRJNA678814) [51]. DNA methylation data generated have been submitted to GEO (GEO accession: GSE161651) [52]. WES sequencing data have been deposited at gated repository [53]; WES dataset requires access control, and there is a corresponding Data Access Committee (DAC) who determines access permissions. Data access requests are reviewed by DAC [53]. Gene expression and DNA methylation data from the TCGA-BLCA cohort were downloaded from UCSC Xena (https://xena.ucsc.edu/), and somatic mutation data and detailed clinicopathological information were obtained from cBioPortal (http://www.cbioportal.org/) under the archive of Bladder Cancer [33]. DNA methylation and RNA-seq expression of bladder cancer cell lines were retrieved from Genomics of Drug Sensitivity in Cancer (GDSC, https://www.cancerrxgene.org/).
