# Supplementary material for: Comprehensive integrative profiling of upper tract urothelial carcinomas
Source: Genome Biol. 2021 Jan 4;22:7. doi: 10.1186/s13059-020-02230-w (PMC7780630; doi:10.1186/s13059-020-02230-w)
Supplement: Supplementary file 2 — Additional file 2: Fig. S1. Copy number variants analysis. Fig. S2. Comparative analysis of the frequency of most frequent mutations in upper-tract urothelial carcinomas (UTUC) as compared to urothelial bladder carcinomas (UBC). Fig. S3. ZFP36 family mutation in diverse histopathological cancer subtypes. Fig. S4. a) Human protein atlas and FANTOM5 datasets showing that protein expression and RNA expression of ZFP36L1 is the highest and the most expressed in the urinary bladder as compared to other tissues. Fig. S5. ZFP36L1 inhibition increases cell motility in vitro. Fig. S6. Distribution of MeTIL score between FGFR3-mutant and wild type of UTUC and UBC, and between muscle-invasive and non-muscle invasive UTUC samples. Fig. S7. Heatmap of BASE47 bladder signature in UTUC samples showing two subgroups of “luminal-like” and “basal-like”. Fig. S8. Principal component analysis showing heterogeneity of the two epi-clusters EpiC-C1 and EpiC-C2, obtained through unsupervised clustering of most variable DNA methylation probes. Fig. S9. Kaplan-Meier curves for progression-free survival regarding UTUC epi-clusters. Fig. S10. Unsupervised clustering of DNA methylation using most variable probes in the whole dataset encompassing UTUC (n = 35) samples and adjacent normal urothelium (n = 8). Fig. S11. Distribution of DNA methylation probes across different genomic regions significantly hypomethylated in FGFR3-mutated tumors versus FGFR3-wild type as compared to EPIC arrays. Heatmap showing differentially methylated probes located in top ranked DMR and corresponding ssGSEA results. Fig. S12. Integrative clustering analysis of multi-omics data. Full Western blots containing the entire ladder for loss-of-function experiments of ZFP36L1 using siRNA in TCCSUP bladder cancer cell line. [file 13059_2020_2230_MOESM2_ESM.docx]

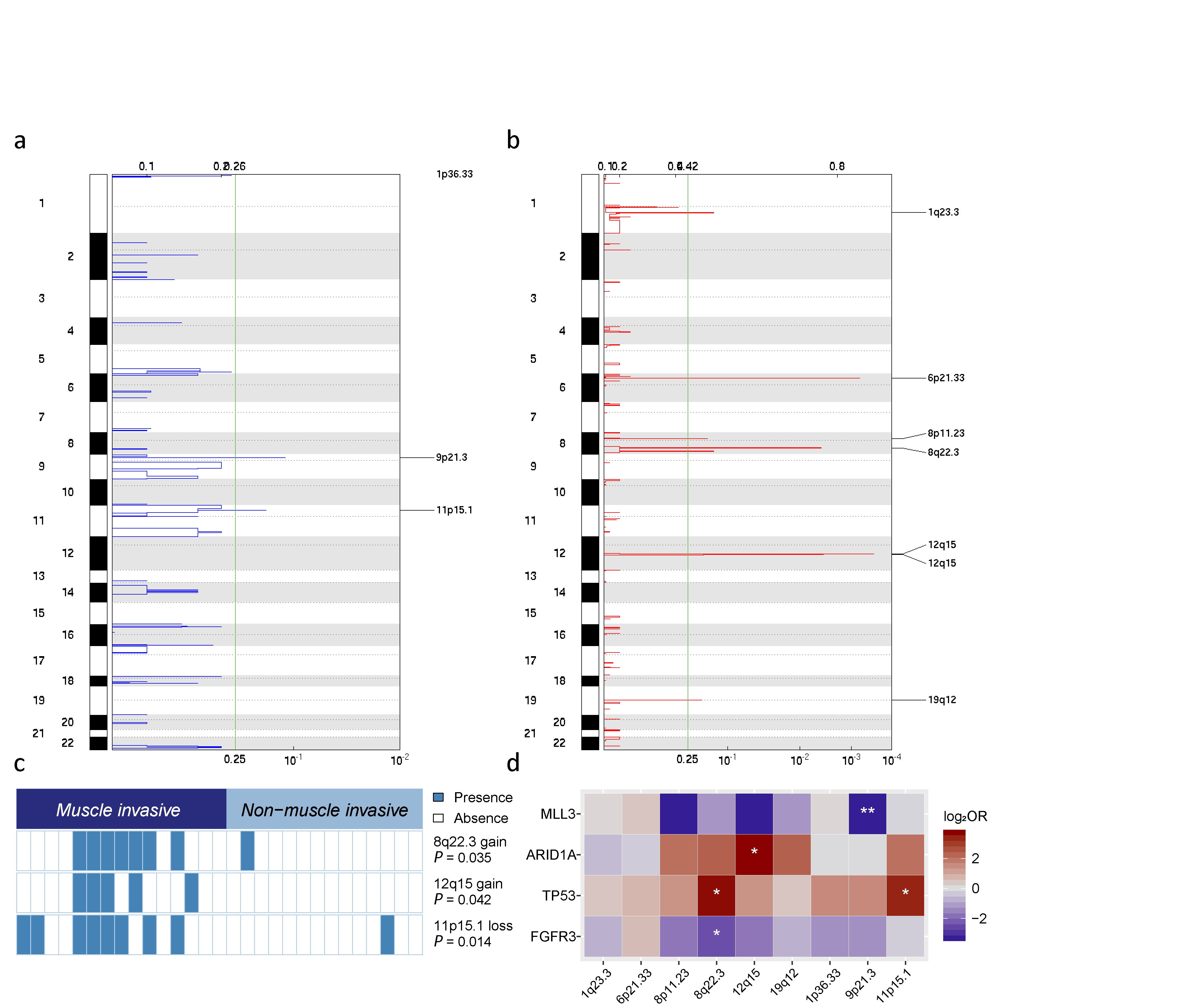


**Fig. S1.** Copy number variants analysis. a-b) GISTIC plot of genomic regions with copy number loss or gain from the exome analysis of the upper-tract urothelial carcinomas. The 21 autosomes are shown on the y axis, and q values indicating statistical significance from the GISTIC analysis are plotted on the x axis for regions of copy number loss (blue at right) and gain (red at left). c) Distribution of significantly enriched GISTIC focal copy number alterations according to muscle-invasive status. d) Heatmap depicting genes and focal copy number alterations with mutual exclusivity or co-occurrence in the whole UTUC cohort. Stars refer to correlations that are statistically significantly.


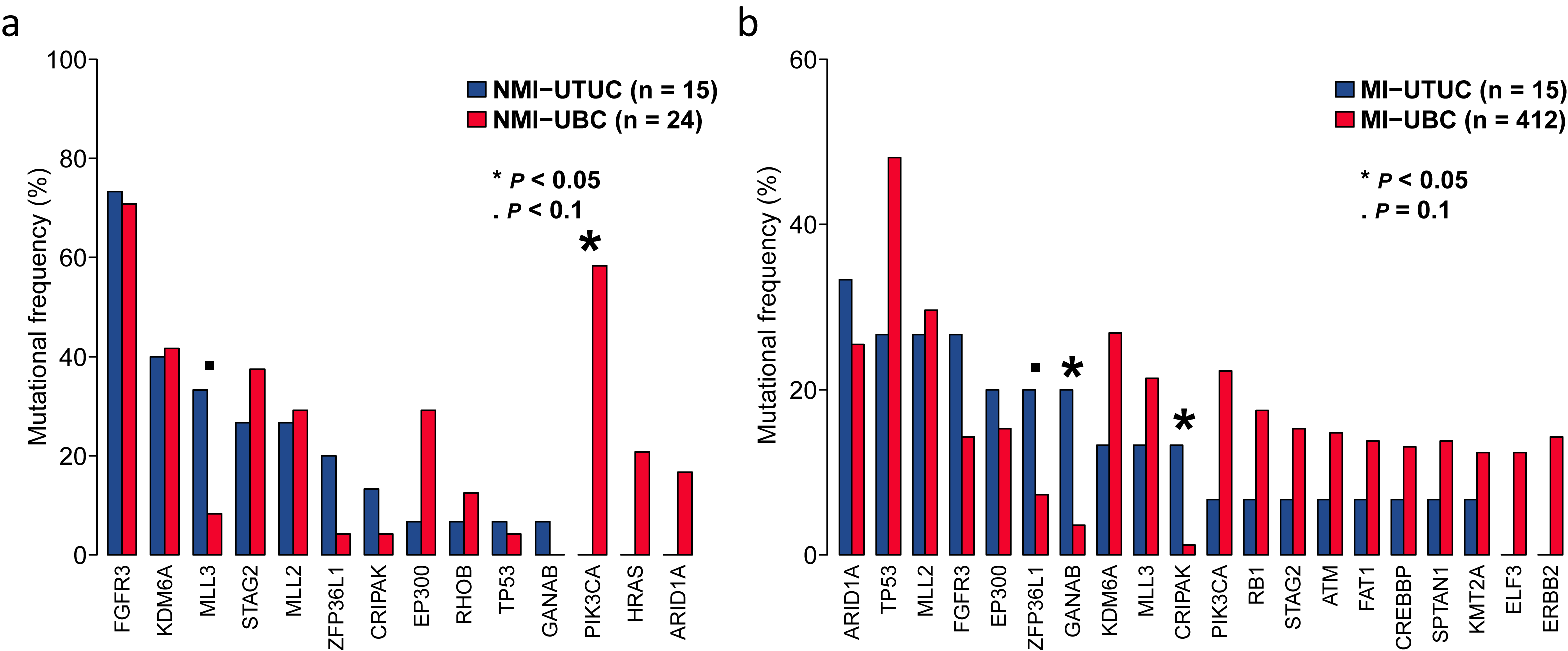


**Fig. S2.** Comparative analysis of the frequency of most frequent mutations in upper-tract urothelial carcinomas (UTUC) as compared to urothelial bladder carcinomas (UBC) for both a) non-muscle invasive samples and b) muscle-invasive samples.


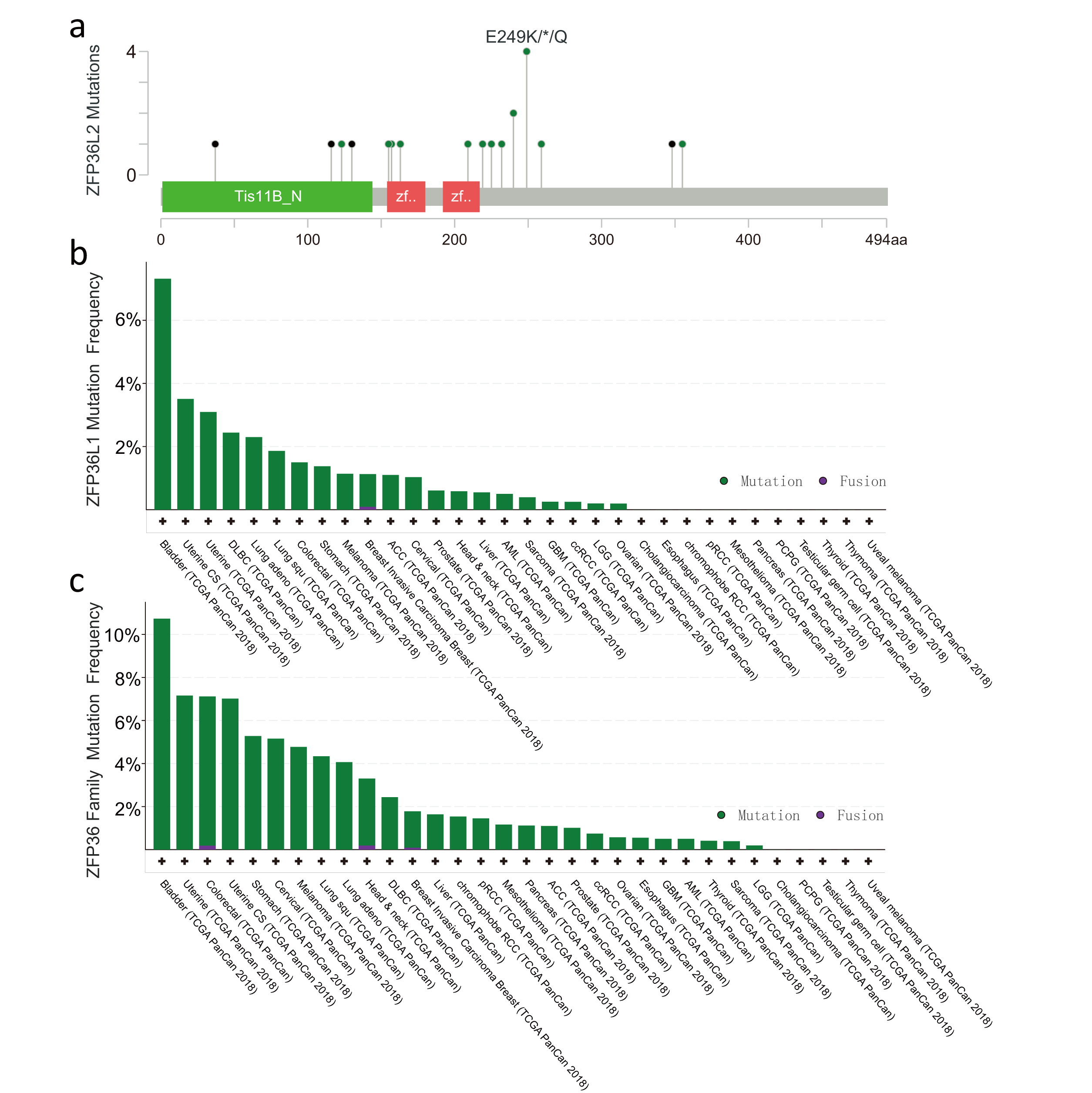


**Fig. S3.** a) Lollipop representing mutations of *ZFP36L2* in TCGA bladder carcinomas cohort with a hotspot mutation occurring at codon E249. b) Frequency of *ZFP36L1* mutations across 10,967 cancer samples related to 32 diverse histopathological cancer subtypes analyzed by TGCA, showing that bladder carcinomas harbored the highest mutational rate. c) Frequency of *ZFP36L1* mutations across 10,967 cancer samples related to 32 diverse histopathological cancer subtypes analyzed by TGCA showing that bladder carcinomas harbored the highest mutational rate.


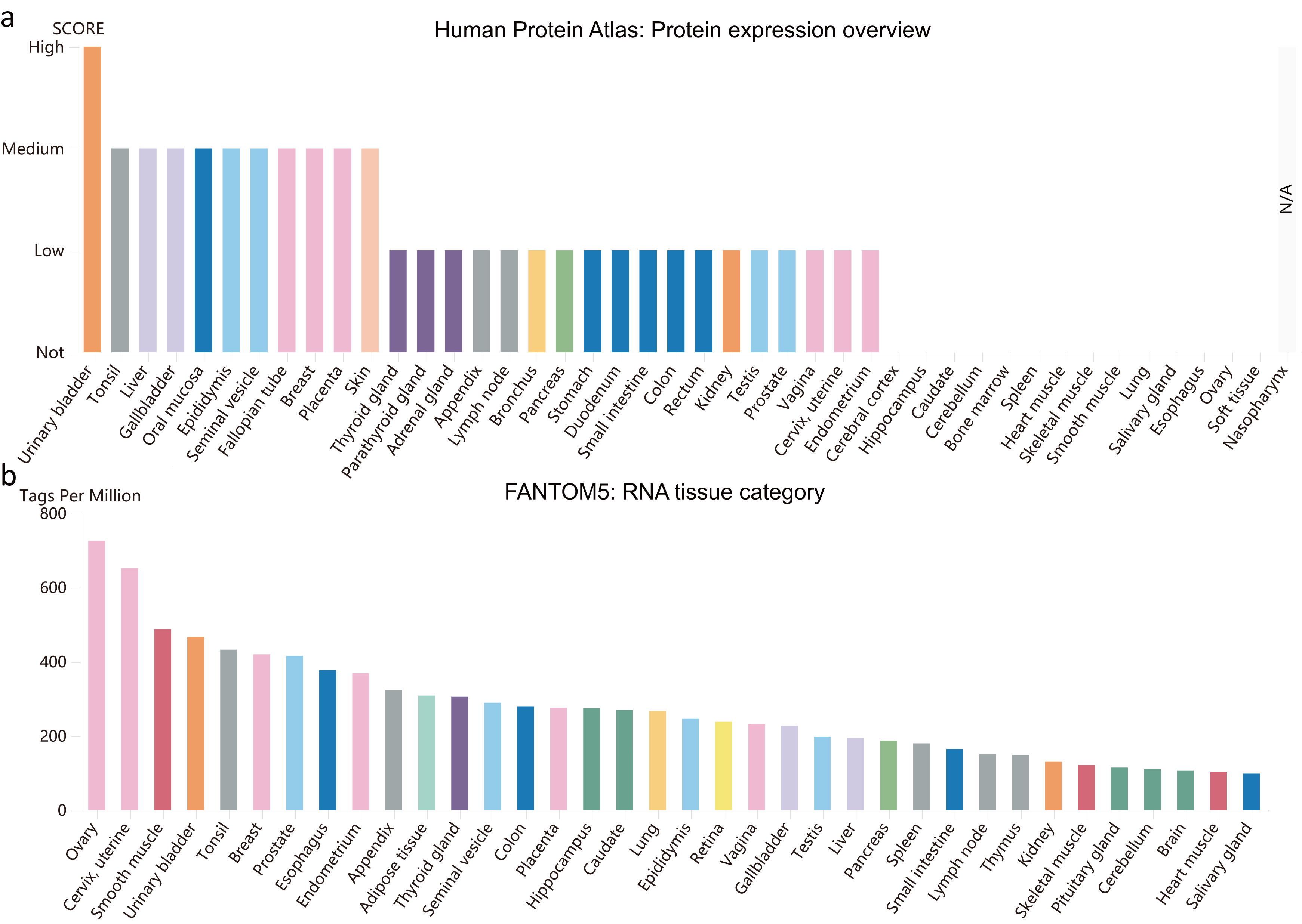


**Fig. S4.** a) Human protein atlas dataset showing that protein expression of *ZFP36L1* is the highest in the urinary bladder as compared to other tissues. b) FANTOM5 showing that RNA expression of *ZFP36L1* is among the most expressed in urinary bladder as compared to other tissues.


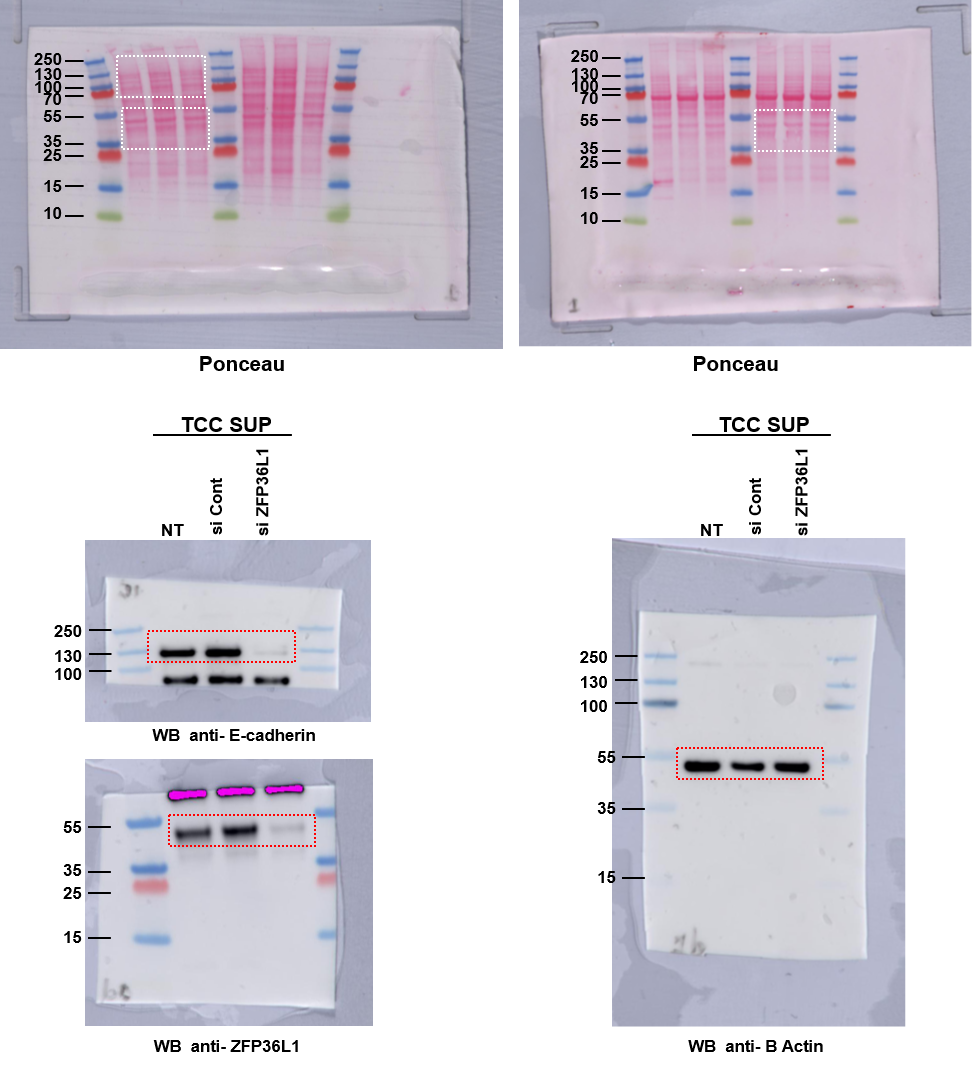


**Fig. S5.** Full Western blots containing the entire ladder for loss-of-function experiments of *ZFP36L1* using siRNA in TCCSUP bladder cancer cell line.


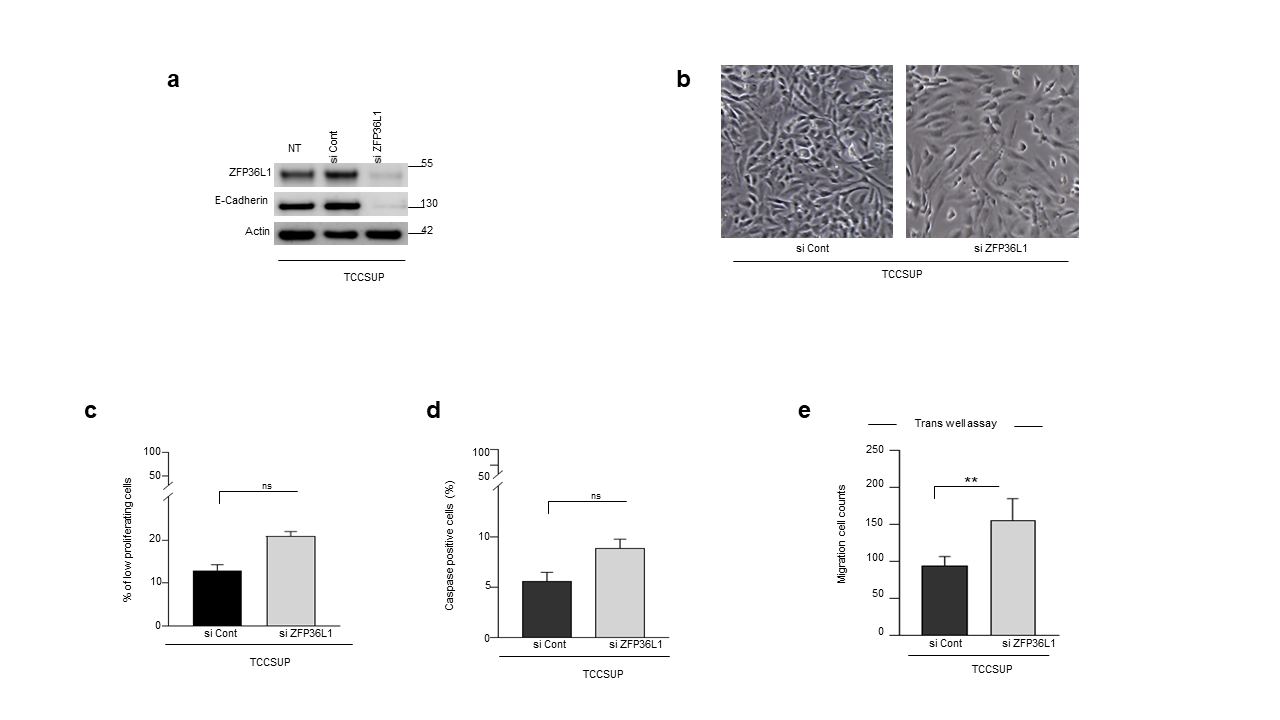


**Fig. S6.** *ZFP36L1* inhibition increases cell motility *in vitro* (a) Western blot analysis of total protein extracts from TCCSSUP cell line non-transfected (NT), transfected with si control (si Cont) and transfected with siRNA targeting *ZFP36L1* (si ZFP36L1). The *ZFP36L1* levels were detected using anti-human *ZFP36L1* antibody and E-Cadherin levels were detected using anti-Cadherin. Actin was used as a loading controls. (b) Cell morphology of TCCSUP cell line transfected with the si Cont (left) and si ZFP36L1 (right) at 10x magnification. Note change of cellular morphology consistent with epithelial-mesenchymal transition (c) Rates of low proliferating cells and (d) caspase positive cells checked in cells after knockdown levels of ZFP36L1 as compared to si Cont cells using flow cytometry. (e) Assessment of migration capability in transwell assay (72h) in cells with knockdown ZFP36L1 levels as compared to the si Cont. The significant differences mentioned above were compared to controls. ns: non-significant. **: *P* < 0.01.


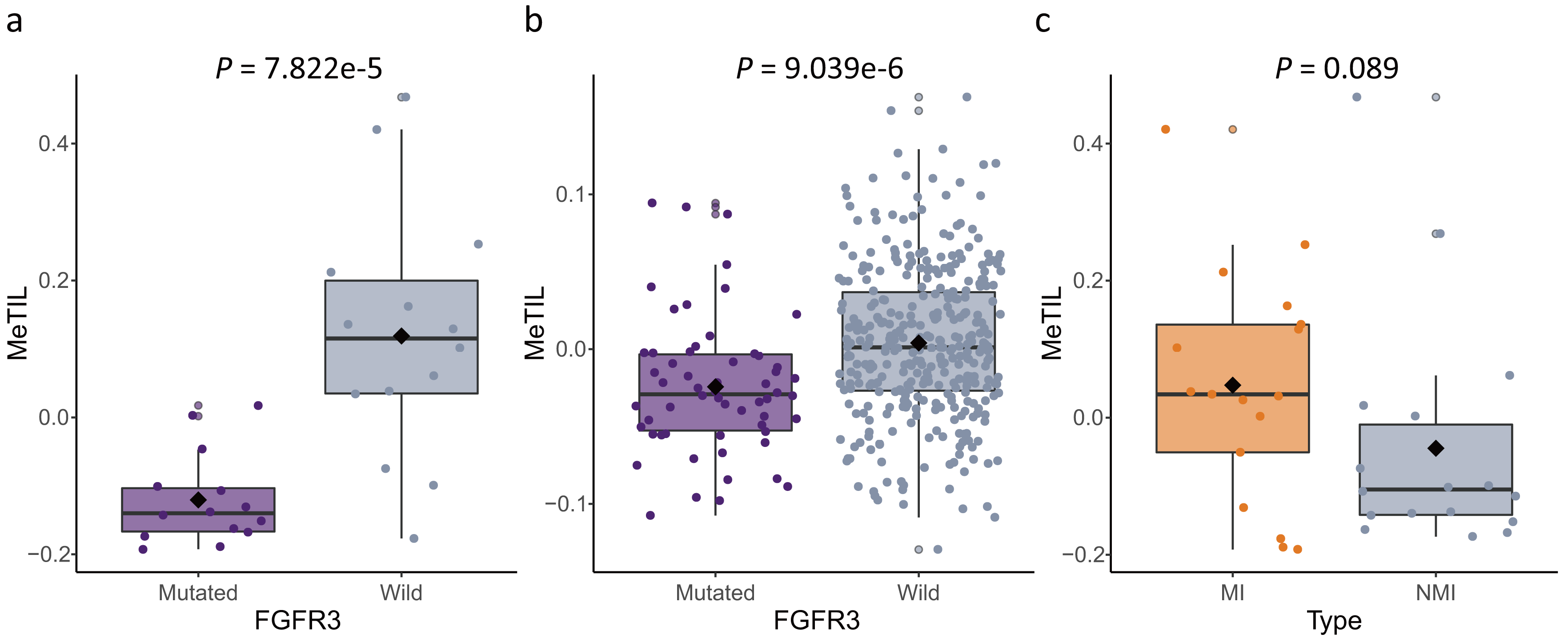


**Fig. S7.** a) Box-plot showing a higher MeTIL score in *FGFR3* wild-type samples as compared to *FGFR3*-mutated UTUC cases. b) Box-plot showing a higher MeTIL score in *FGFR3* wild-type samples as compared to *FGFR3*-mutated BLCA cases in the TCGA bladder cohort. c) Box-plot showing a tendency toward higher MeTIL scores in muscle-invasive (MI) as compared to non-muscle invasive (NMI) UTUC cases.


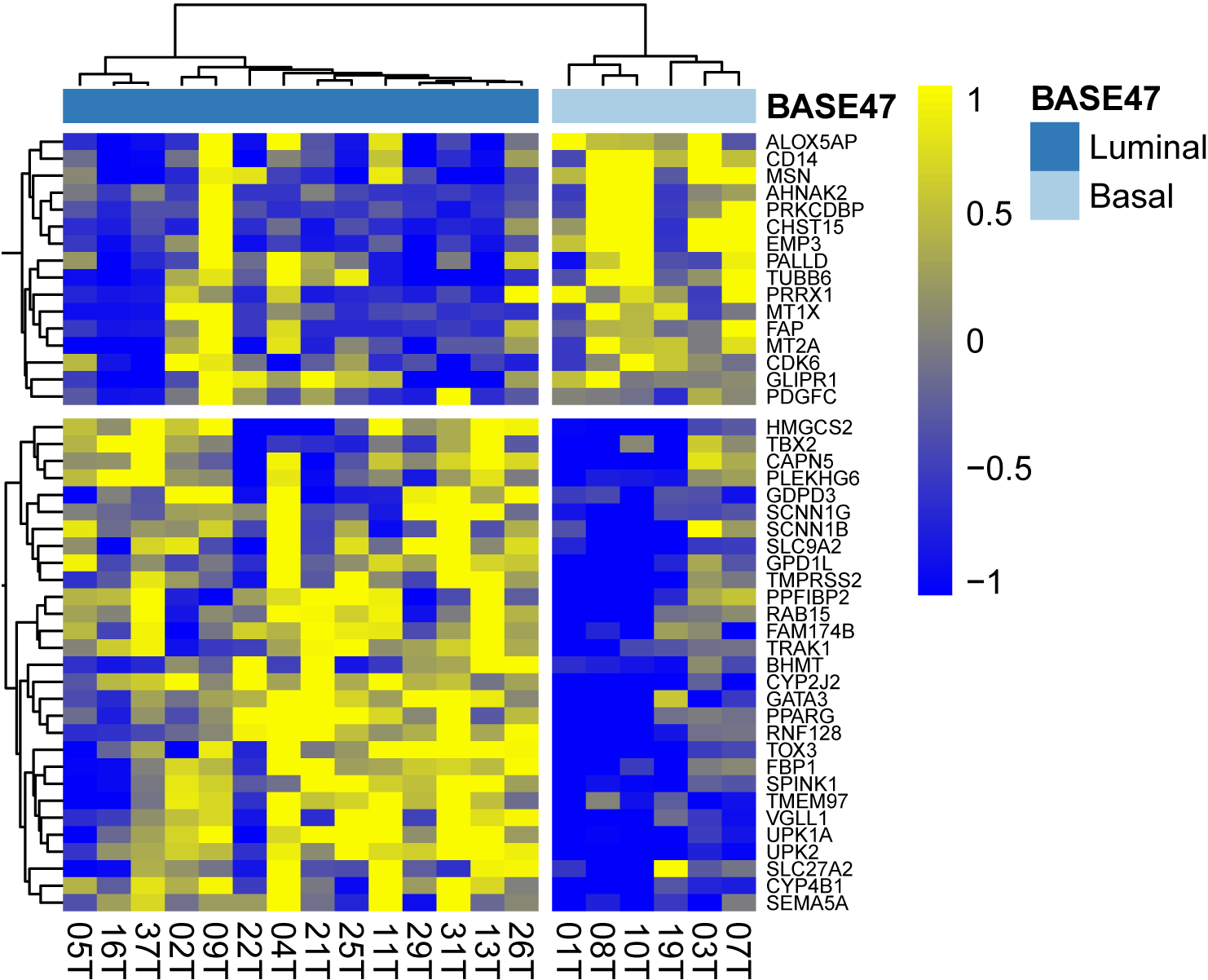


**Fig. S8.** Heatmap of BASE47 bladder signature in UTUC samples showing two subgroups of “luminal-like” and “basal-like”.


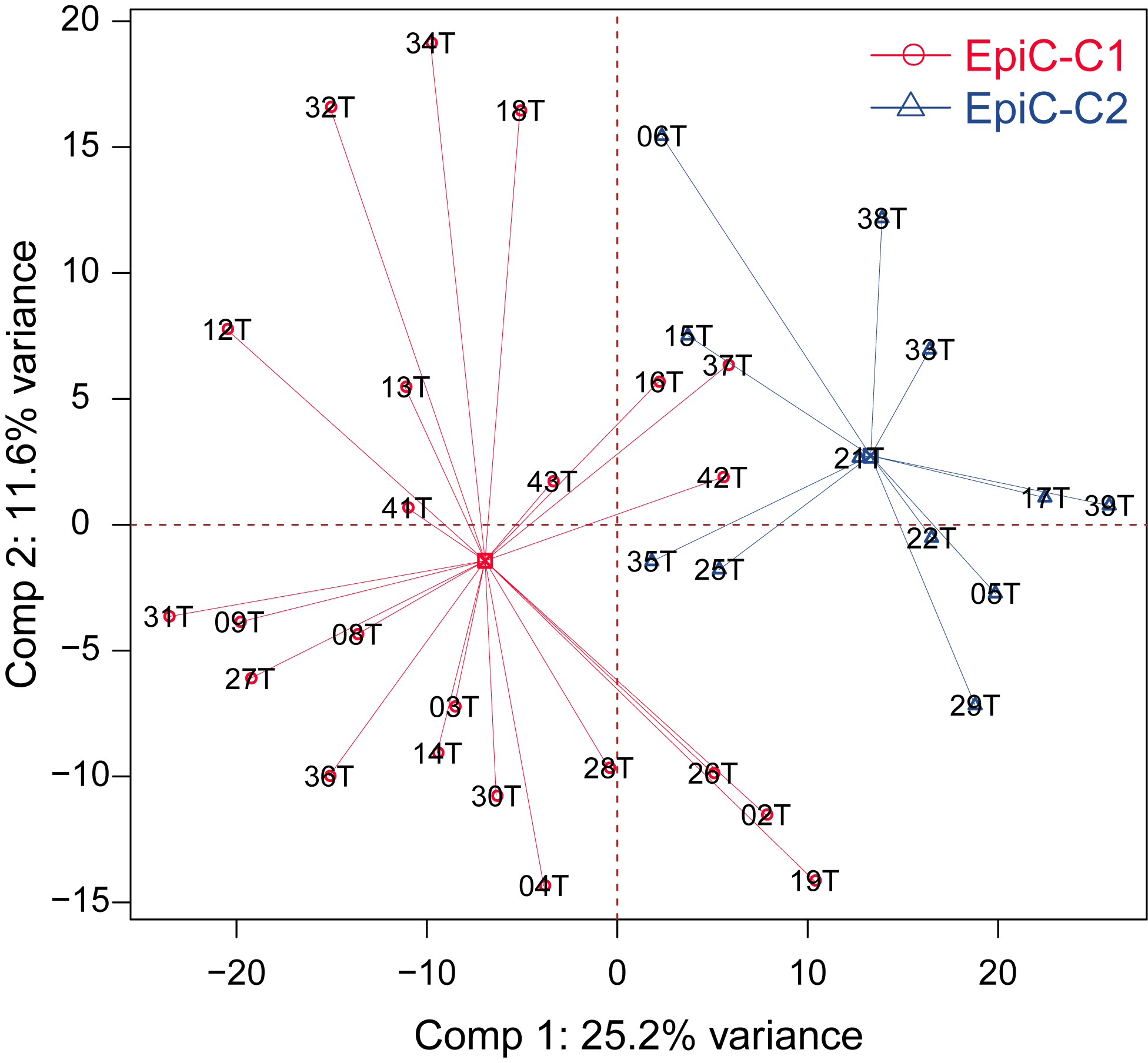


**Fig. S9.** Principal component analysis showing heterogeneity of the two epi-clusters EpiC-C1 and EpiC-C2, obtained through unsupervised clustering of most variable DNA methylation probes.


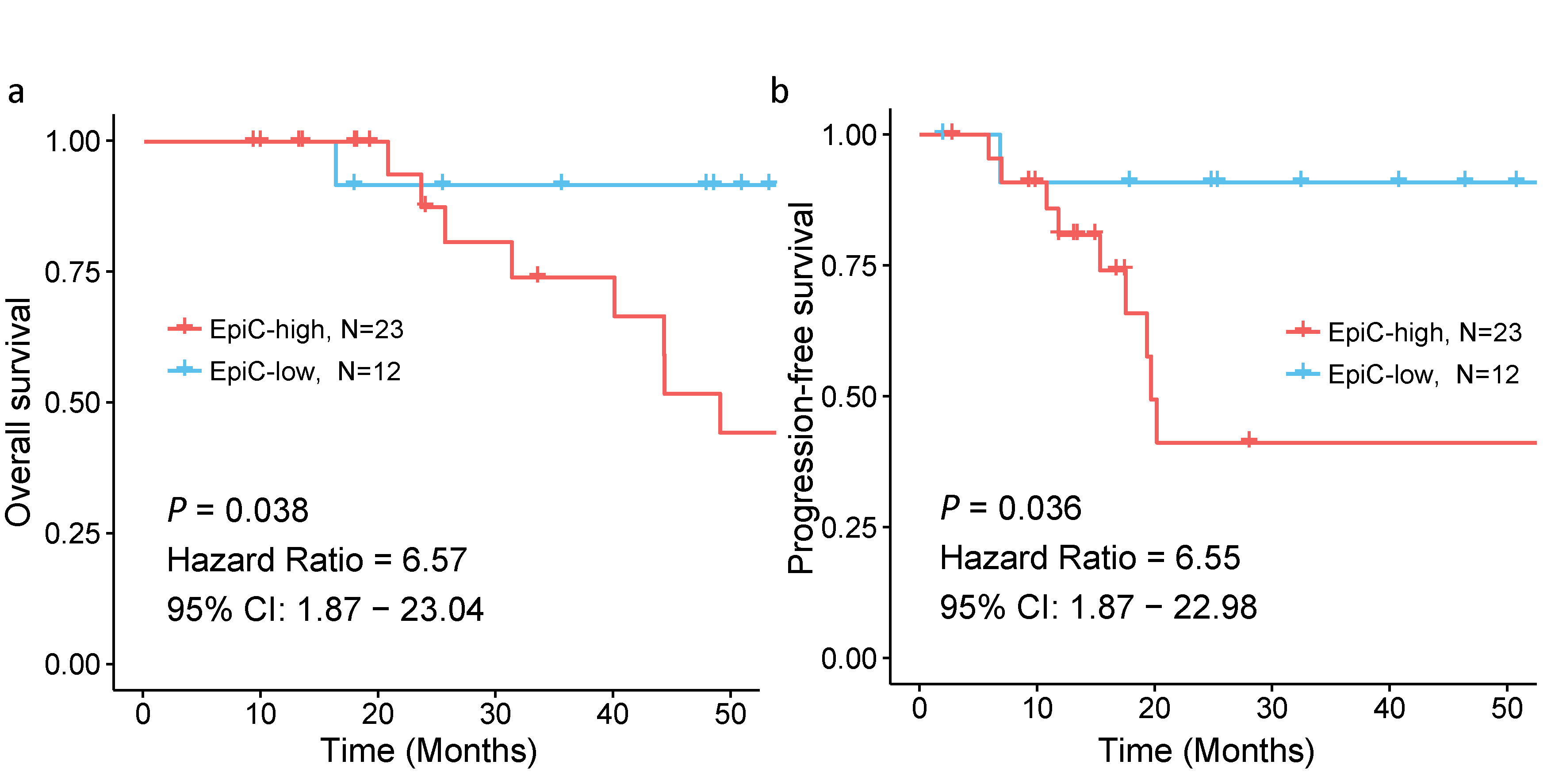


**Fig. S10.** Kaplan-Meier curves for progression-free survival (a) and overall survival (b) according to the DNA methylation based classification in EpiC-high and EpiC-low UTUC subtypes.


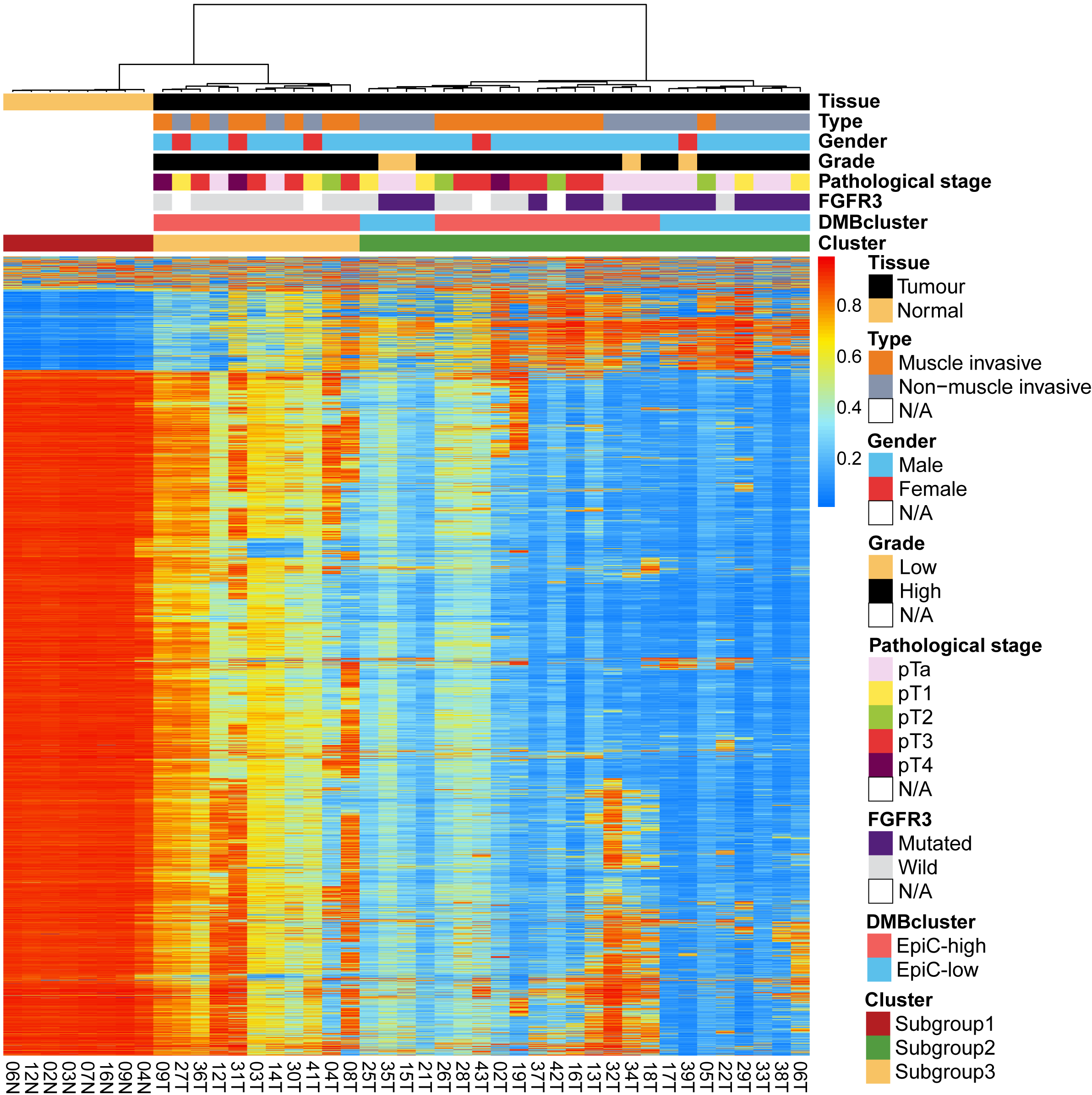


**Fig. S11.** Unsupervised clustering of DNA methylation using most variable probes in the whole dataset encompassing UTUC (n = 35) samples and adjacent normal urothelium (n = 8). Note that all normal samples gather together and that UTUC samples are composed of two subgroups associated with *FGFR3* mutations.


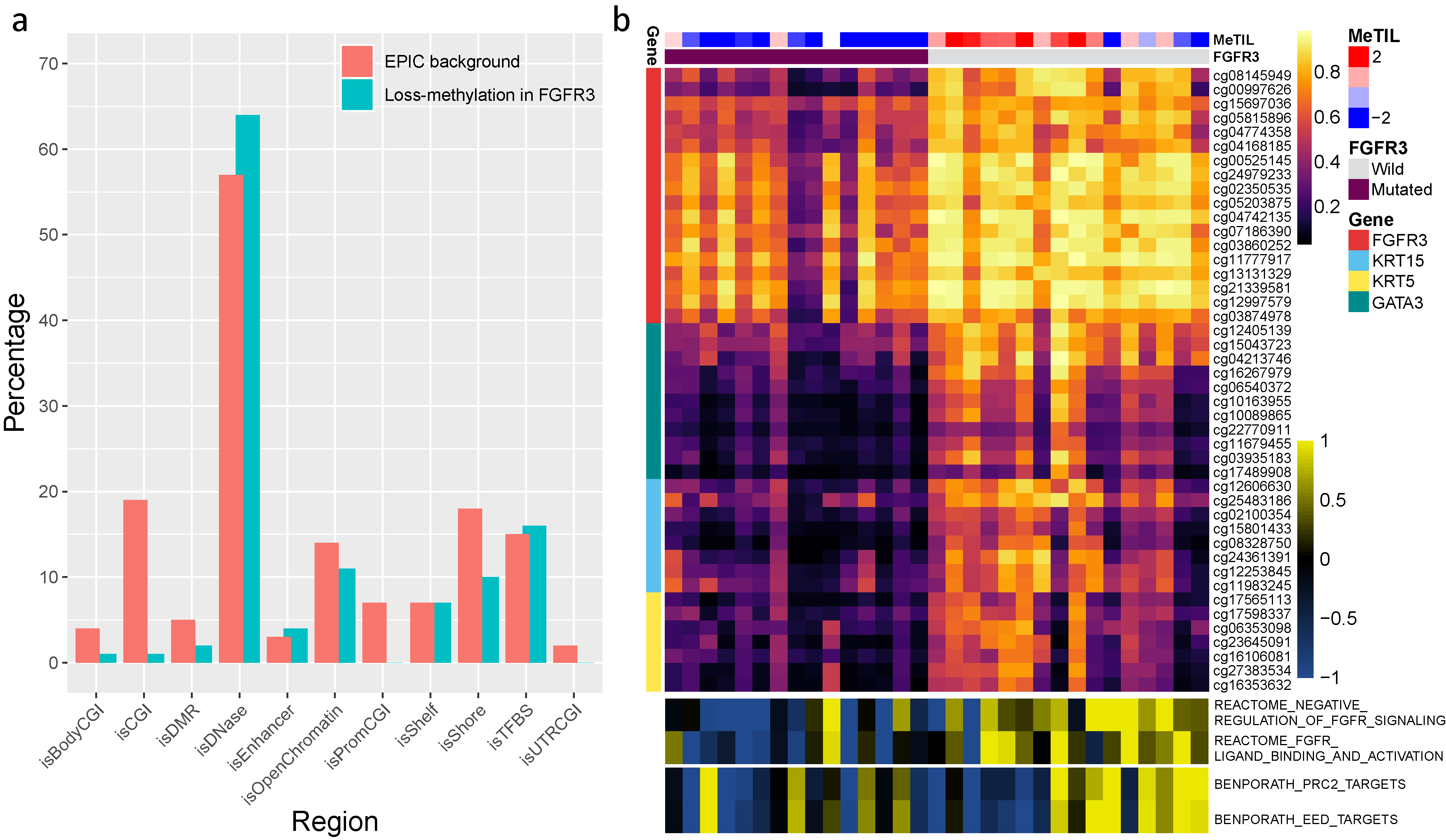


**Fig. S12.** a) Percentage and distribution of DNA methylation probes across different genomic regions significantly hypomethylated in *FGFR3*-mutated tumors versus *FGFR3*-wild type (blue) as compared to EPIC arrays (red). Note that there is no enrichment identified according to different genomic regions annotated by EPIC arrays. b) Heatmap showing differentially methylated probes located in top ranked differentially methylated regions (DMR), including genes known to be involved in bladder cancer. The bottom part depicts results of single-sample Gene Set Enrichment Analysis (GSEA) for those DMR.


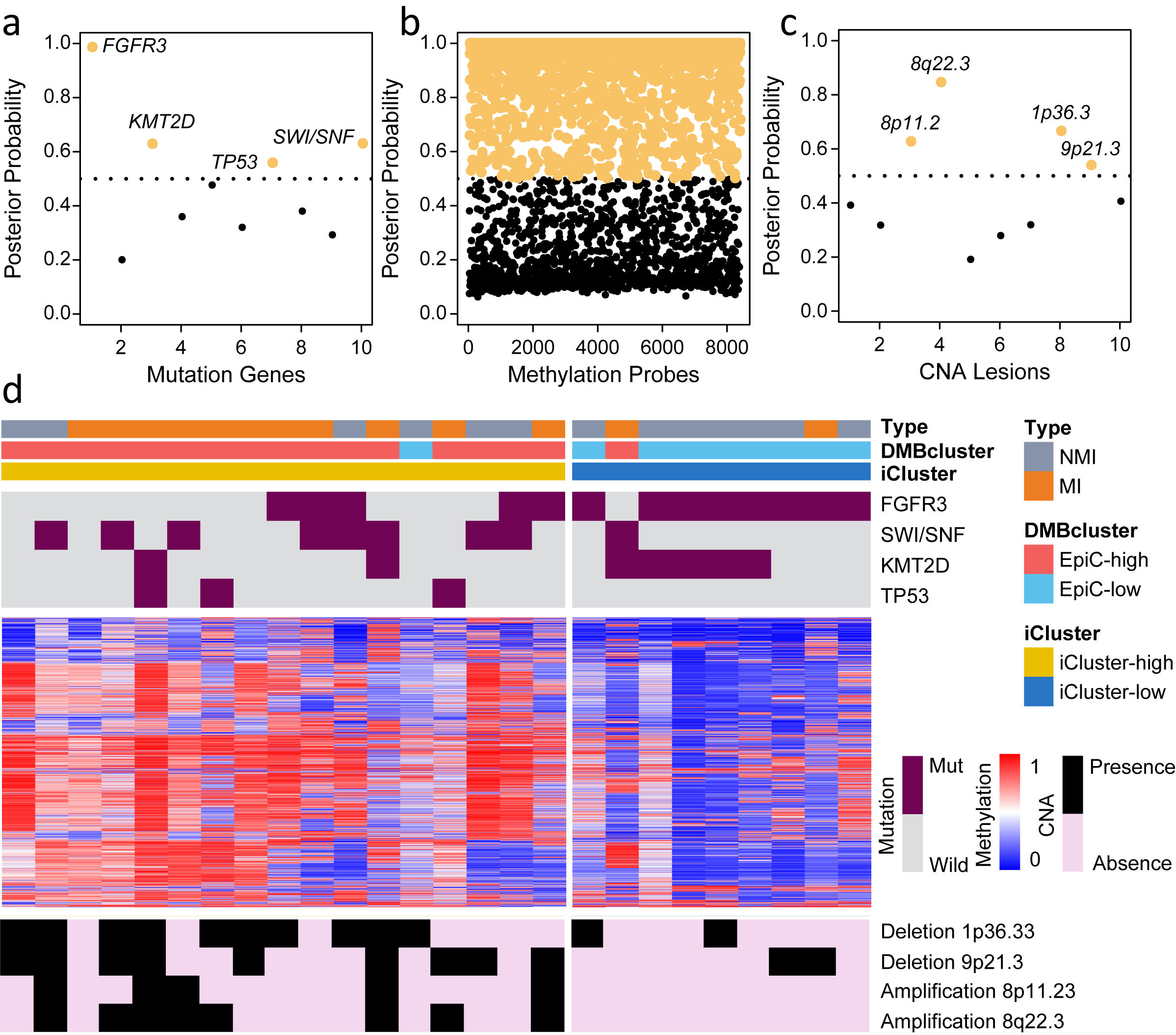


**Fig. S13.** Integrative clustering analysis of multi-omics data. a-c) Bayesian Integrative clustering (iClustering) analysis for somatic mutations, differentially methylated probes, and somatic copy-number variation. d) iClustering defining two subgroups of UTUC: iCluster-low and iCluster-high. UTUC iCluster-low is enriched for non-muscle invasive UTUC with *FGFR3* and *KMT2D* mutations, while iCluster-high showed a tendency to be enriched for *SWI/SNF* mutations and 8q22.3 gain.
