# Supplementary material for: Comprehensive integrative profiling of upper tract urothelial carcinomas
Source: Genome Biol. 2021 Jan 4;22:7. doi: 10.1186/s13059-020-02230-w (PMC7780630; doi:10.1186/s13059-020-02230-w)
Supplement: Supplementary file 3 — Additional file 3. Review history (LOG 180 bytes). [file 13059_2020_2230_MOESM3_ESM.docx]

Review History

**First round of review**

**Reviewer 1**

**Are you able to assess all statistics in the manuscript, including the appropriateness of statistical tests used?** Yes, and I have assessed the statistics in my report.

**Comments to author:**

The authors described a multi-omics study on upper-tract urothelial carcinomas (UTUC), including DNA SNV and CNV, transcriptional signatures and epigenetic DNA methylation profiling of UTUC. First, they identified a novel frequently mutated gene ZFP36L1 in UTUC. Second, according to these omics data, they developed multiple classifiers to subtype UTUC, such as MWBcluter, RNAcluster and DMBcluster. Third, they showed that the EpiC-low subtype tumors are hypomethylated, enriched for FGFR3-mutated, and have a better prognosis. However, there are several comprehensive genomic and transcriptional studies on UTUC have been done. For example, in order to investigate the intrinsic biology diversity between UTUC and urothelial bladder cancer, John et al performed targeted sequencing of 300 cancer associated genes in 83 UTUC and 102 UCB samples and demonstrated that FGFR3 (35.6%), HRAS (136%) and CDKN2B (15.3%) are more frequently altered in high-grade UTUC (doi: 10.1016/j.eururo.2015.07.039). Tyler et al performed WES, RNAseq and protein analysis of 31 untreated UTUC samples, they identified frequent gene mutations in FGFR3 (74.1%), KMT2D/MLL2 (44.4%), PIK3CA (25.9%) and TP53 (22.2%), they also defined 4 molecular subtypes based on RANseq data (doi: 10.1016/j.eururo.2017.05.048). Moreover, Francois et al performed targeted next generation sequencing on DNA samples of 195 UTUC and 454 UCB and found that FGFR3 and HRAS are more frequently mutated in UTUC rather than UBC (doi: 10.1158/1078-0432.CCR-18-2039). More recently, Xuesong et al performed whole-genome sequencing of 90 UTUC tumors and defined 3 subtypes with prognostic relevance (doi: https://doi.org/10.1101/735324).

Therefore, compared to these published studies, the sample size (30 samples for WES genomic analysis, 20 samples for transcriptional analysis, 35 samples for methylome) of this study is relatively small. Also, most of the mutated genes identified in this study have been reported previously in large cohort of UTUC samples, including FGFR3, KDM6A, KMT2D/MLL2, KMT2C/MLL3, STAG2, ARID1A and TP53. Overall the manuscript provided some new findings, however the contents of the paper remains descriptive without clear connection and functional validation. In my view, there are five major issues need to be strengthened in order for this to become an important contribution:

Major comments:

1, ZFP36L1 was identified a novel frequently mutated gene in UTUC, and most are truncated mutations, suggesting that ZFP36L1 might function as a tumor suppressor in UTUC. However, the authors showed that mutations of ZFP36 family genes are not associated with clinicopathological features and overall survival of UTUC patients. Also, the authors presented that urothelial bladder cancers with ZFP36 family gene mutations are associated with a better overall survival based on TCGA data, which imply that ZFP36 family mutations might be a protective event in urothelial bladder cancer. Thus, the clinical relevance of ZFP36L1 mutation in UTUC is limited. Moreover, the biological roles of ZFP36L1 loss-of-function mutation in UTUC are still unknown? The authors are asked to provide functional experimental results to address these issues.

2, this manuscript provided multi-omics data, including WES SNV and CNA alterations, RNAseq transcriptional changes and DNA methylation profiling. However, only the DNA methylome data presented here are novel to the field of urothelial cancer research. The authors first used genomic mutation profiles and transcriptional expression profiling to define molecular subtypes of UTUC, respectively. However, both subtypes (MWBcluter and RNAcluster) are not associated with any clinicopathological features or TIL. The clinical relevance of these two classifiers is limited.

For the DNA methylation classification (DMBcluster), about 1/3 samples in EpiC-high group have relatively hypomethylation probes (figure 5a), there is no clear two distinct subtypes based on the DNA methylation profiling. Also, this study found that the EpiC-low subtype contains most of the FGFR3-mutated samples, tumors with low pathological stages and also non-muscle invasive tumors, therefore, patients belong to the EpiC-low subtype have a better overall survival. However, FGFR3 mutations have been demonstrated to be associated with better survival of UTUC and bladder cancers (https://doi.org/10.1016/j.eururo.2008.06.013). As expected, patients with non-muscle invasive tumors also have a better prognosis compared with muscle invasive tumor. Therefore, the clinical significance and potential application of this DMBcluster is limited.

3, this study hypothesized that FGFR3 mutation might be associated with disticnt epigenentic alterations, since EpiC-low subtpye tumors are FGFR3-mutated. However, these findings are still descriptive. The authors showed that no statistically significant enriched promoter was found between FGFR3-mutant and WT tumor, or MI and NMI tumors. It will be worth to investigate whether FGFR3 mutation can directly regulate global DNA methylation in UTUC. If FGFR3 inhibitors can remodel the global DNA methylation changes of UTUC tumors ? Also, this study proposed that there are correlations between methylation status and immune cells infiltration or SWI/SNF gene mutations. However, the authors did not provide further experimental results to confirm these findings.

4, this manuscript presented too much contents (Figure 6 and Figure 7) on bladder cancer based on TCGA data. In particular, the authors just validated the DMBcluster in bladder MI TCGA samples and got siminlar results as these in UTUC, such as FGFR3-mutated tumors are also hypomethylated and have better overall survival in bladder cancers. Would be better to focus on identifying intrinsic differences between UTUC and UBC cancers based on the data generated in the current study.

5, this study also developed an iCluster classifier, iCluster-high and iCluster-low, by integrative clustering of CNV, mutation and methylation data. However, this iCluster almost overlaps with the DMBcluster. What is the clinical significance of this iCluster subtype, If the iCluster has a better power to predict overall survival of UTUC patients. The authors should take advantage of these multi-omics data to identify candidate "drivers" in UTUC by integrative analysis of SNV, CNV, transcriptional and methylation profiling.

Other comments:

typo on page 14 line 46, BLCA-C2 should be BLCA-C1.

**Reviewer 2**

**Are you able to assess all statistics in the manuscript, including the appropriateness of statistical tests used?**  No, I do not feel adequately qualified to assess the statistics.

**Comments to author:**

Upper-tract urothelial carcinoma (UTUC), which is deadly at the muscle-invasive stage, is a relatively rare cancer to which very few -omics studies have been devoted.

The work of Su et al. provides new information about UTUC and urothelial carcinomas in general. However, some parts could be shortened, particularly the DNA methylation analyses.

Main points

This study provides the first convincing evidence for a role of the ZFP36 family genes as tumor suppressors in cancer, particularly in UTUC, but also in bladder cancer.

- Intriguingly, the pattern of mutations reported in a previous -omics study on UTUC (Robinson et al., Nat Commun 2019) differs markedly from that reported here. Robinson et al. identified KMT2D as the most frequently mutated gene in this cancer (35% of cases) (not found in Su et al.), and found no mutation of ZFP36L1 (20% in Su et al.).

The existence of two classes based on DNA methylation (named "epicluster low" and "epicluster high" by the authors), with different molecular and clinical features, is of potential interest in terms of tumor biology and may have clinical implications.

- In my opinion, the methylation analyses reported in Figure 6 add little to the work and could be deleted. Furthermore, the results of these analyses are not reported by the authors in the abstract or discussed.

- It is intriguing that most (all but one) of the muscle-invasive UTUCs belong to epicluster high whereas a much more even distribution is reported for muscle-invasive bladder cancer (n=219 for epicluster high and n=192 for epicluster low).

- Taking normal samples into account, the authors provide an additional tumor classification, also into two tumor clusters, based on DNA methylation (Figure S8), which appears to be very different from the epicluster high/epicluster low classification.

Other remarks

- FGFR3 mutations were studied in 36 tumors (Table S1). However, the survival data according to FGFR3 mutations reported include findings for only 30 tumors (Figure S1).

- Should the terms "highly altered" be used (page 9) in cases of 6-8.5% mutation?

- The authors compare their classification with previous molecular classifications of bladder cancer. However, they use different classifications in different parts of the manuscript (in Figure S6, they use TCGA subtypes, whereas, in Figure 4, they use the subtypes of the consensus classification).

- The conclusions drawn from Figure 4b and Figure S6 are quite different. In Figure 4b, most of the UTUCs are closer to the luminal papillary class, whereas, in Figure S6, the UTUCs are closer to the luminal infiltrated class (possibly related to the point above?).

- Only 20 tumors were studied by RNA-seq, but it would have been interesting to know whether this different groups identified by the different approaches are related (transcriptome clusters or assignment based on previous classification, signature clusters, epiclusters).

- Figure 1b, the y axis should be labeled.

- Figure 2d,e, the scale should be the same.

- Several of the survival curves reported in the supplementary figures are of little interest as they simply reflect the relationship between the parameters used to separate the samples and disease stage.

**Authors Response**

**Point-by-point responses to the reviewers’ comments:**

**Reviewer #1:** **General comments :** The authors described a multi-omics study on upper-tract urothelial carcinomas (UTUC), including DNA SNV and CNV, transcriptional signatures and epigenetic DNA methylation profiling of UTUC. First, they identified a novel frequently mutated gene ZFP36L1 in UTUC. Second, according to these omics data, they developed multiple classifiers to subtype UTUC, such as MWBcluter, RNAcluster and DMBcluster. Third, they showed that the EpiC-low subtype tumors are hypomethylated, enriched for FGFR3-mutated, and have a better prognosis. However, there are several comprehensive genomic and transcriptional studies on UTUC have been done. For example, in order to investigate the intrinsic biology diversity between UTUC and urothelial bladder cancer, John et al performed targeted sequencing of 300 cancer associated genes in 83 UTUC and 102 UCB samples and demonstrated that FGFR3 (35.6%), HRAS (136%) and CDKN2B (15.3%) are more frequently altered in high-grade UTUC (doi: 10.1016/j.eururo.2015.07.039). Tyler et al. performed WES, RNAseq and protein analysis of 31 untreated UTUC samples, they identified frequent gene mutations in FGFR3 (74.1%), KMT2D/MLL2 (44.4%), PIK3CA (25.9%) and TP53 (22.2%), they also defined 4 molecular subtypes based on RANseq data (doi: 10.1016/j.eururo.2017.05.048). Moreover, Francois et al performed targeted next generation sequencing on DNA samples of 195 UTUC and 454 UCB and found that FGFR3 and HRAS are more frequently mutated in UTUC rather than UBC (doi: 10.1158/1078-0432.CCR-18-2039). More recently, Xuesong et al performed whole-genome sequencing of 90 UTUC tumors and defined 3 subtypes with prognostic relevance (doi: <https://doi.org/10.1101/735324>).
Therefore, compared to these published studies, the sample size (30 samples for WES genomic analysis, 20 samples for transcriptional analysis, 35 samples for methylome) of this study is relatively small. Also, most of the mutated genes identified in this study have been reported previously in large cohort of UTUC samples, including FGFR3, KDM6A, KMT2D/MLL2, KMT2C/MLL3, STAG2, ARID1A and TP53. Overall the manuscript provided some new findings, however the contents of the paper remains descriptive without clear connection and functional validation. In my view, there are five major issues need to be strengthened in order for this to become an important contribution:
**General answer : We would like to thank reviewer 1 for his thorough evaluation for our work. We agree with him that there have been several reports investigating genomic alterations of UTUC, however, the majority of them were based on targeted sequencing as mentioned (Audenet et al ; Skafianos et al), except for Tyler and Robinson studies which we acknowedged in our manuscript. In addition, we reported for the first time DNA methylation landscape of muscle-invasive and non-muscle invasive UTUC and correlated our findings with genetic and transcriptomic tumor features. Our main discovery is the identification of *ZFP36L1* as novel recurrently mutated genes in UTUC and we provided herein in the revised version a functional study regarding its role in TCCSUP cell line. In addition, we now provided novel data clearly showing that bladder cancer cell lines with *FGFR3* alterations harbor similar DNA methylation as *FGFR3* mutated UTUC samples. Thus, we believe that the current version is highly improved and would like to thank reviewer 1 for his suggestions and remarks.**

**Major comments:**

**Comment 1 :** ZFP36L1 was identified a novel frequently mutated gene in UTUC, and most are truncated mutations, suggesting that ZFP36L1 might function as a tumor suppressor in UTUC. However, the authors showed that mutations of ZFP36 family genes are not associated with clinicopathological features and overall survival of UTUC patients. Also, the authors presented that urothelial bladder cancers with ZFP36 family gene mutations are associated with a better overall survival based on TCGA data, which imply that ZFP36 family mutations might be a protective event in urothelial bladder cancer. Thus, the clinical relevance of ZFP36L1 mutation in UTUC is limited. Moreover, the biological roles of ZFP36L1 loss-of-function mutation in UTUC are still unknown? The authors are asked to provide functional experimental results to address these issues.

**Answer to comment 1 : We would like to thank reviewer 1 for his comments. To adress the question of biological role of *ZFP36L1*, we herein performed an additional loss of function experiments of *ZFP36L1* in TCCSUP cell line and showed increased migration of cells using transwell assay; notably, this was associated with E-Cadherin loss, consistent with epithelial-mesenchymal transition (EMT). No effect was observed for cell proliferation and apoptosis. A new section has now been added in the main text as well as a new figure (Figure S5). We also added a new section in the discussion commeting on the results observed. These data are consistent with previous loss of functions experiments (Loh X. et al. (DOI: 10.1158/0008-5472.CAN-18-2796) using different bladder cancer cell lines and demonstrating that silencing of *ZFP36L1* mildly enhanced tumor cell growth and foci formation, while its forced expression in cancer cells markedly reduced cell proliferation and migration through reduction of cell motility-genes (vimentin and N-cadherin). Further experiments are thus need to explain the mechanim.** **Collectively, these findings demonstrated the role of *ZFP36L1* as a tumor suppressor in urothelial carcinomas. We expanded now on describing these findings in the discussion part of the manuscript.**

**Figure S5 : *ZFP36L1* inhibition increases cell motility *in vitro* (a) Western blot analysis of total protein extracts from TCCSSUP cell line non-transfected (NT), transfected with si control (si Cont) and transfected with siRNA targeting *ZFP36L1* (si ZFP36L1). The *ZFP36L1* levels were detected using anti-human *ZFP36L1* antibody and E-Cadherin levels were detected using anti-Cadherin. Actin was used as a loading controls. (b) Cell morphology of TCCSUP cell line transfected with the si Cont (left) and si ZFP36L1 (right) at 10x magnification**. **Note change of cellular morphology consistent with epithelial-mesenchymal transition** **(c) Rates of low proliferating cells and (d) caspase positive cells checked in cells after knockdown levels of ZFP36L1 as compared to si Cont cells using flow cytometry. (e) Assessment of migration capability in transwell assay (72h) in cells with knockdown ZFP36L1 levels as compared to the si Cont. The significant differences mentioned above were compared to controls. ns: non-significant. **: *P* < 0.01.**

**Comment 2 :** this manuscript provided multi-omics data, including WES SNV and CNA alterations, RNAseq transcriptional changes and DNA methylation profiling. However, only the DNA methylome data presented here are novel to the field of urothelial cancer research. The authors first used genomic mutation profiles and transcriptional expression profiling to define molecular subtypes of UTUC, respectively. However, both subtypes (MWBcluter and RNAcluster) are not associated with any clinicopathological features or TIL. The clinical relevance of these two classifiers is limited.
For the DNA methylation classification (DMBcluster), about 1/3 samples in EpiC-high group have relatively hypomethylation probes (Figure 5a), there is no clear two distinct subtypes based on the DNA methylation profiling. Also, this study found that the EpiC-low subtype contains most of the *FGFR3*-mutated samples, tumors with low pathological stages and also non-muscle invasive tumors, therefore, patients belong to the EpiC-low subtype have a better overall survival. However, *FGFR3* mutations have been demonstrated to be associated with better survival of UTUC and bladder cancers (<https://doi.org/10.1016/j.eururo.2008.06.013>). As expected, patients with non-muscle invasive tumors also have a better prognosis compared with muscle invasive tumor. Therefore, the clinical significance and potential application of this DMBcluster is limited.

**Answer to comment 2 : We would like to thank reviewer 1 for his important remarks. The purpose of our analysis was to define the added value of DNA methylation to genetic and transcriptome sequencing in dissecting molecular underpinnings of NMI and MI UTUC. We agree that our findings do not support that genetic and transcriptome classification add much to predict patients outcome, probably due to limited number size. However, to most of our knowledge, and as mentioned by reviewer 1, the DNA methylation analysis is novel to UTUC and to urothelial carcinomas field, as we are the first to report on the methylome of MI and NMI UTUC. Our data clearly showed that while usual clinico-pathological tumor features were not associated with outcome in our cohort, DNA methylation classification was able to predict both disease-free survival and overall survival, suggesting the importance of epigenetic landscapes in defining patients outcome. One possible explanation for the absence of significant difference for DFS and OS between NMI and MI cases in our cohort is the low number of cases. To better strenghten the importance of our findings, we have now added a new figure (Figure 5g) with forest-plots of DFS and OS showing that classical clinical features were not able to predict outcome in our cohort in contrast to our DMB classification.**

**Regarding DNA methylation clustering and to obtain subtype classifications of UTUC samples (n = 35), we performed unsupervised hierarchical clustering using the 1% most variable probes, after excluding probes with no available values and those located on sex chromosomes (n = 836,691). We identified two robust DNA methylation epi-clusters: EpiC-C1 (n = 23 ; 65.7%) and EpiC-C2 (n=12; 34.3%); heterogeneity within two clusters is now supported by Principal component analysis (Figure S8) which we now added in the manuscript. To determine the differentially methylated probes (DMPs) and differentially methylated regions (DMRs) between the two identified epi-clusters, we harnessed the chip analysis methylation pipeline (ChAMP) using default parameters of EPIC arrays. Using the most stringent criteria, a total of 14,243 significantly hypermethylated probes were identified from the background of 242,687 DMPs. Among these, EpiC-C1 presented frequent hypermethylation, since 99.8% (14,209) of the probes were significantly hypermethylated as compared to EpiC-C2 in which only 34 probes gained methylation. In this manner, we redesignate EpiC-C1 as EpiC-high and EpiC-C2 as EpiC-low accordingly. Thus, we believe that our DNA methylation signature might be of great clinical relevance as it outperforms classical clinical features used to predict patients outcome and wich were not relevant in our small cohort. Thus, larger cohorts are needed to confirm our observation.**

**Comment 3 :** This study hypothesized that FGFR3 mutation might be associated with disticnt epigenentic alterations, since EpiC-low subtpye tumors are FGFR3-mutated. However, these findings are still descriptive. The authors showed that no statistically significant enriched promoter was found between FGFR3-mutant and WT tumor, or MI and NMI tumors It will be worth to investigate whether FGFR3 mutation can directly regulate global DNA methylation in UTUC. If FGFR3 inhibitors can remodel the global DNA methylation changes of UTUC tumors ? Also, this study proposed that there are correlations between methylation status and immune cells infiltration or SWI/SNF gene. mutations. However, the authors did not provide further experimental results to confirm these findings.

**Answer to comment 3 : We would like to thank reviewer 3 for his excellent comment. Although, we did not observe statistically significant enrichment for DNA methylation in promoters located in CpG islands between *FGFR3*-mutant and WT tumors, or between MI and NMI tumors, we strikingly observed dramatic changes in DNA methylation outside CpG islands. Although these data are not causative, and do not demonstrate mechanistically that *FGFR3* remodel the DNA methylome, they highly indicate that epigenetic changes in *FGFR3* mutant tumors do not occur randomly. Thus, the question whether *FGFR3* mutation can directly or indirectly regulate global DNA methylation in UTUC is of great interest. We thus decided to analyse DNA methylation of 20 bladder cancer cell lines for which DNA methylation data was available (450K). Notably, supervised clustering using the 14,209 hypermethylated probes derived from UTUC EpiC-high vs EpiC-low, identified two subgroups tighthly associated with *FGFR3* status (Figure 6a). Notably, all but one cell line (DSH1) in C2 cluster have *FGFR3* fusions with no SWI/SNF gene mutations. Conversly, 2 out of 16 cell lines in C1 cluster displayed *FGFR3* mutations; notably, *FGFR3* mutation is known to be non functional in J82 cell lines with no expression of *FGFR3*, and *FGFR3* mutations in 639V cell line co-occured with *SMARCA4*. In addition, supervised clustering of *FGFR3* gene expression signature showed similar findings (expression data was available for 18 cases) (Figure 6b). Although, not causative, these data suggest that *FGFR3* may remodel directly or indirectly the methylome. Future mechanistic data are thus needed to better understand mechanistically if the epigenetic remodeling occur directly or indirectly. A new section with a new Figure 6 (below) has now been added in the main text summarizing our new findings.**

**Regarding the link between immunity and genetic alterations, consistent with our UTUC data, we observed similar findings in urothelial cancer cell lines as *FGFR3* altered cases (C2) showed lower signature of IFN-gamma genes relative to C1 (*P*=0.022) (Figure 6c). However, whether this is specific to SWI/SNF mutations is unclear. Further studies are needed to clarify this observation, especially that published mechanistic studies exploring the link between mutations in SWI/SNF and immunity are contradictory. The discussion has been also modified to take in account our findings as well as the contradictory results of the literature.**

**Figure 6. a) Supervised clustering using hypermethylated probes derived from UTUC EpiC-high vs EpiC-low clusters and showing two subgroups of bladder cancer cell lines linked with SWI/SNF mutations (C1) and *FGFR3* translocation status (C2). Note that the probes depicted in the heatmap were those that were statistically significant. b) Supervised clustering extracted from Mahe of bladder cancer cell lines according to *FGFR3* gene expression signature (using RT112 bladder cancer cell line). Note that although DSH1 cell line do not harbor any *FGFR3* mutation, it clusters with the three other cell lines harboring *FGFR3* fusions and share similar transcriptomic program. c) Box-plots for IFN-gamma scores using single-sample Gene Set Enrichment Analysis (ssGSEA) in C1 and C2 clusters.**

**Comment 4 :** this manuscript presented too much contents (Figure 6 and Figure 7) on bladder cancer based on TCGA data. In particular, the authors just validated the DMBcluster in bladder MI TCGA samples and got similar results as these in UTUC, such as *FGFR3*-mutated tumors are also hypomethylated and have better overall survival in bladder cancers. Would be better to focus on identifying intrinsic differences between UTUC and UBC cancers based on the data generated in the current study.

**Answer to comment 4: we would like to thank the reviewer 1 for his suggestion. To comply with his suggestion, we removed original Figure 6 from our revised version. In addition, we analyzed difference between genetic and transciptomic alterations of UTUC and bladder carcinomas. For this purpose, results of somatic mutations of NMI (n=15) and MI (n=15) UTUC were compared to NMI Hurst et al. cohort (n=24) and MI TCGA (n=412) cohorts, respectively (Figure S2). We observed increased frequency of *PIK3CA* in NMI bladder as compared to NMI UTUC (*P*=0.0001). In addition, we observed increased rate of *GANAB* (*P*=0.021) and *CRIPAK* (*P*=0.022) mutations in MI UTUC as compared to MI bladder carcinomas, with tendency toward higher rate of *ZFP36L1* mutations (*P*=0.1). Regarding gene expression results, we herein showed that bladder UTUC are similar to luminal papillary urothelial carcinomas, with few cases belonging to other urothelial carcinomas subtypes. We also performed clustering of gene expression using BASE47 signature (Damrauer JS et al. PNAS, 2014). We observed that the majority of our cases were luminal-like  (n=14), while 6 cases were classified as basal-like. Figure 4a has been updated to refect the results of different classifications already reported.**

**Figure S2 : Comparative analysis of the frequency of most frequent mutations in upper-tract urothelial carcinomas as compared to ureothelial bladder carcinomas (UBC) for both a) non-muscle invasive samples and b) muscle-invasive samples.**

**Figure S7 : Heatmap of BASE47 bladder signature in UTUC samples showing two subgroups of “luminal-like” and “basal-like”.**

**Comment 5 :** this study also developed an iCluster classifier, iCluster-high and iCluster-low, by integrative clustering of CNV, mutation and methylation data. However, this iCluster almost overlaps with the DMBcluster. What is the clinical significance of this iCluster subtype, If the iCluster has a better power to predict overall survival of UTUC patients. The authors should take advantage of these multi-omics data to identify candidate "drivers" in UTUC by integrative analysis of SNV, CNV, transcriptional and methylation profiling.

**Answer to comment 5 : The goal of integrative clustering in general is indeed to take account for multi-omics data. In UTUC, we agree with the reviewer that the results overlap with DMB epi-clusters, suggesting the importance of the crosstalk between epigenetic alterations from one side and genetic alterations and gene expression from the other side. To comply with reviewers comment, we analyze survival according to the iCluster classification. The iClusters cannot seperate either PFS (*P*=0.313) or OS (*P*=0.283) because of the relatively small sample size of 26 (2 out of 28 has no CNA data). Because the idea for jointly clustering is to assume there is a crosstalk between different data type, so by identifying iClusters we connect mutations, CNA and methylation to find that *FGFR3* mutations co-occur with DNA hypomethylation.**

**Other comments:**

typo on page 14 line 46, BLCA-C2 should be BLCA-C1.

**Response : Thanks for poiting this out. This has been corrected.**

**Reviewer #2 comments :**

**General comments :** Upper-tract urothelial carcinoma (UTUC), which is deadly at the muscle-invasive stage, is a relatively rare cancer to which very few -omics studies have been devoted.
The work of Su et al. provides new information about UTUC and urothelial carcinomas in general. However, some parts could be shortened, particularly the DNA methylation analyses.

**General answers : We would like to thank reviewer 2 for this comments. We shortened DNA methylation analysis by removing the section regarding the integration of the data with bladder TCGA. Figure 6 has been removed also.**

**Main points :**

**Comment 1 :** This study provides the first convincing evidence for a role of the ZFP36 family genes as tumor suppressors in cancer, particularly in UTUC, but also in bladder cancer.
Intriguingly, the pattern of mutations reported in a previous -omics study on UTUC (Robinson et al., Nat Commun 2019) differs markedly from that reported here. Robinson et al. identified KMT2D as the most frequently mutated gene in this cancer (35% of cases) (not found, in Su et al.) and found no mutation of ZFP36L1 (20% in Su et al.).

**Answer to comment 1: We would like to thank reviewer 2 for his nice comments. Thanks for pointing out differences between Robinson et al. cohort and the current one, probably due to the nature of UTUC samples included. While Robinson et al. study did not include low-grade UTUC samples and was composed primarly from MI UTUC (n=23 ; 62.2%), our cohort included 50% of MI UTUC (n=15) and 13.3% of low grade tumors (n=4) which might explain the slight differences. The prevalence of *KMT2D/MLL2* and *KMT2C/MLL3* mutations rate were 27% and 23% in our cohort, as compared to 35.1% and 8.1% in Robinson et al. study. To avoid confusion regarding gene annotations, we changed the text annotations of *MLL2* and *MLL3* to *KMT2D/MLL2* and *KMT2C/MLL3*, respectively.**

**Comment 2 :** The existence of two classes based on DNA methylation (named "epicluster low" and "epicluster high" by the authors), with different molecular and clinical features, is of potential interest in terms of tumor biology and may have clinical implications. In my opinion, the methylation analyses reported in Figure 6 add little to the work and could be deleted. Furthermore, the results of these analyses are not reported by the authors in the abstract or discussed

**Answer to comment 2 : we would like to thank reviewer 2 for his comment. Methylation analysis in Figure 6 have been removed.**

**Comment 3 :** It is intriguing that most (all but one) of the muscle-invasive UTUCs belong to epicluster high whereas a much more even distribution is reported for muscle-invasive bladder cancer (n=219 for epicluster high and n=192 for epicluster low).

**Answer to comment 3. We totally agree with this observation. Several mechanisms might lead to *FGFR3* activation in muscle-invasive bladder carcinomas, which might explain these results. In a recent analysis, 73% of bladder carcinomas samples with *FGFR3* mutations showed *FGFR3* overexpression versus 22% among *FGFR3* wild-type tumor (**[**Bas W G van Rhijn**](https://pubmed.ncbi.nlm.nih.gov/?sort=date&term=van+Rhijn+BWG&cauthor_id=32682615) **et al. European Urology, 2020). Furthere studies are needed to clarify if there is a link between *FGFR3* overexpression (without *FGFR3* mutations) and methylome remodeling.**

**Comment 4 :** Taking normal samples into account, the authors provide an additional tumor classification, also into two tumor clusters, based on DNA methylation (Figure S8), which appears to be very different from the epicluster high/epicluster low classification.

**Answer to comment 4 : We agree that there is a difference using normal samples. However, the results are consistent with the notion that *FGFR3* mutated samples are hypomethylated as compared to their normal urothelial counterparts. Indeed, none of the samples belonging to subgroup 2 had *FGFR3* mutations (n=0/9), as compared to 68.2% (n=15/22) in subgroup 3 (*P*=0.0001). As for the majority of studies, cancer subtypes classification are often performed using cancer samples, we thought that including this analysis would be important to show specifically that UTUC samples are hypomethylated globaly as compared to normal samples.**

**Other remarks :**

- FGFR3 mutations were studied in 36 tumors (Table S1). However, the survival data according to FGFR3 mutations reported include findings for only 30 tumors (Figure S1).

**Response : We herein report the data for 30 samples as those are related to the cases for which WES sequencing was done. Analysis using 36 samples showed consistent tendencies. Figure S1 has been removed as suggested below.**

- Should the terms "highly altered" be used (page 9) in cases of 6-8.5% mutation?

**Response : We agree to temper our claim and this has been changed to "altered".**

- The authors compare their classification with previous molecular classifications of bladder cancer. However, they use different classifications in different parts of the manuscript (in Figure S6, they use TCGA subtypes, whereas, in Figure 4, they use the subtypes of the consensus classification). The conclusions drawn from Figure 4b and Figure S6 are quite different. In Figure 4b, most of the UTUCs are closer to the luminal papillary class, whereas, in Figure S6, the UTUCs are closer to the luminal infiltrated class (possibly related to the point above?). Only 20 tumors were studied by RNA-Seq, but it would have been interesting to know whether this different groups identified by the different approaches are related (transcriptome clusters or assignment based on previous classification, signature clusters, epiclusters).

**Response : We agree with the reviewer with the points raised. To avoid diluting the message, we decided to keep the Figure 4b showing that majority of UTUC has luminal papillary class. Original Figure S6 has been removed. In addition, we performed BASE47 bladder cancer classification (Figure S7) and showed that 14 UTUC samples were classified as "luminal-like" and 6 samples as "basal-like". We then integrated the annotations of BASE47 classification, consensus classification as well as our own transcriptomic, genetic and epigenetic classification in the same figure (Figure 4a). Interestingly, the 6 cases classified as basal using BASE47 were classified as basal/squamous (n=2), stroma-rich (n=2) and luminal papillary (n=2) using consensus classification. Figure 4a has been changed accordingly.**

**Figure 4a : Unsupervised clustering of UTUC samples. Bladder consensus classification, BASE47 classification, DMB clusters and MWB clusters were added. Abbreviations : DMBcluster: DNA methylation based epi-cluster; MWBcluster: Mutation weight based cluster; RNAcluster: RNA expression based cluster.**

- Figure 1b, the y axis should be labeled.

**Response : Thank you for this considerate concern, and we have modified Figure 1b.**

- Figure 2d,e, the scale should be the same.

**Response : Sorry for this confusion, and we have modified Figure 2d and e.**

- Several of the survival curves reported in the supplementary figures are of little interest as they simply reflect the relationship between the parameters used to separate the samples and disease stage.

**Response : We agree with reviewer 2. Supplementary figure 1 has been removed.**

**Second round of review**

**Reviewer 1**

This revised manuscript has improved a lot. The authors address most of my concerns. Overall, the findings of this study will be interested to field of urothelial cancers.

**Reviewer 2**

The authors have responded satisfactorily to my reviews
